# Microbial community multi-omic analysis of marsh sediment following enrichment with crustacean shell compost

**DOI:** 10.1093/ismeco/ycag307

**Published:** 2026-10-24

**Authors:** Colleen E Yancey, Kyle D Brumfield, Laurence Ettwiller, Rita R Colwell

**Affiliations:** Research Division, New England Biolabs Inc., Ipswich, MA 01938, United States; University of Maryland Institute for Advanced Computer Studies, University of Maryland, College Park, MD 20740, United States; Department of Cell Biology and Molecular Genetics, University of Maryland, College Park, MD 20740, United States; Maryland Pathogen Research Institute, University of Maryland, College Park, MD 20740, United States; Research Division, New England Biolabs Inc., Ipswich, MA 01938, United States; University of Maryland Institute for Advanced Computer Studies, University of Maryland, College Park, MD 20740, United States; Department of Cell Biology and Molecular Genetics, University of Maryland, College Park, MD 20740, United States; Maryland Pathogen Research Institute, University of Maryland, College Park, MD 20740, United States

**Keywords:** coastal environments, multi-omics, microbial ecology, pathogens, salt marsh

## Abstract

Nutrient availability influences microbial processes in natural environments, notably biogeochemical cycling and community structure. Short-term, functional responses of microbial communities to nutrient pulses in coastal communities are not fully understood, especially in temperate environments undergoing climate driven changes. A 48 hour microcosm experiment was carried out in which paired metagenomic and metatranscriptomic sequencing was employed to elucidate how decomposition of chitin rich substrates, namely, crab and lobster shell compost, alter salt marsh microbiome structure and function. Within 48 hours of amendment, a notable shift in community function was observed, including increased chitin degradation, altered stress-response, and enhanced sporulation. These responses coincided with marked decreases in genes regulating key biogeochemical processes, including carbon fixation, sulfur oxidation and reduction, and other metabolic pathways. After addition of shell compost, changes in microbial community structure and increased incidence of virulence-associated genes were also observed, accompanied by measurable transcriptional activation, notably aerolysin A *(aerA)*, which encodes the pore-forming exotoxin aerolysin. These results support shifts in both community composition and function in temperate salt marsh sediment microbiomes following chitin-rich nutrient addition. It is concluded that temperate coastal systems are sensitive to organic nutrient enrichment, with the potential for ecological and public-health relevant consequences. These effects may be particularly significant given chitin is among the most abundant and readily available bionutrients in aquatic ecosystems.

## Introduction

Coastal environments, including salt marshes and estuaries, provide several ecosystem services including biogeochemical cycling, fishery nurseries, aquaculture, and buffer zones during extreme precipitation events [1, 2]. These coastal systems harbor largely uncharacterized microbial diversity, significant in biogeochemical cycling of carbon, nitrogen, and sulfur [3–5]. Of note is the importance of salt marshes in carbon turnover and sequestration, largely driven by microbial communities inhabiting these environments [5, 6]. However, while these microbial communities provide a variety of services benefiting both ecosystem health and society, they may also harbor potentially harmful bacteria [7–10].

Intensification of extreme events and related climatic shifts subject marine environments to increased coastal flooding, rising temperatures, and outbreaks of disease [11–14]. These events fundamentally reshape the structure of coastal ecosystems, with implications for public and environmental health. Sea level rise and storm intensification are driving widespread degradation and loss of salt marshes, reducing their ability to buffer the impact of storms leading to amplification of flooding and ecosystem instability [15, 16]. These changes promote the emergence and spread of pathogens, increasing the frequency and severity of disease in marine organisms, including commercially important crustaceans, and humans [13, 17–20]. While autochthonous potential pathogens, notably *Vibrio* spp., are generally present in low abundance in aquatic ecosystems, their proliferation can be exacerbated during hurricanes and extreme flooding events [7, 21–23].

Chitin, the second most abundant natural polymer in the world, is a bioavailable polysaccharide found in the exoskeleton of crustaceans, insects, and fungi. Chitin plays an important role in both the carbon and nitrogen biogeochemical cycling of marine systems where it serves as a nutrient-rich substrate for a variety of microbial taxa [24, 25], including bacteria colonizing shellfish and crustaceans [19, 26–28]. Salt marshes, critical buffer zones during flooding, are vulnerable to nutrient loading, namely, organic matter accumulation during extreme weather events [29] where microbial growth is accelerated, and opportunistic pathogens emerge [21].

The Great Marsh spans the North Shore of Massachusetts and the New Hampshire Seacoast, encompassing over 20 000 acres. Like many such systems globally, it is subject to coastal inundation with more frequent coastal flooding and salt marsh restructuring [30, 31]. Sediments in this system are relatively rich in organic carbon compared to other eastern US salt marshes [32], suggesting efficient accumulation and accretion. Yet, long-term carbon storage in the sediment is declining, as eutrophication reduces root biomass and organic matter accumulation, resulting in the loss of marsh elevation [33]. Large-scale crustacean die-offs have also been documented in Southern New England, with warming-driven disease incidence, including epizootic shell disease, increasingly affecting Gulf of Maine lobster populations [34–36]. The Great Marsh provided a unique site to determine the response of microbial population dynamics to temperate system nutrient pulses and declining carbon sequestration in a changing climate.

This study focused on short-term pulse disturbances of chitin-rich detritus in temperate salt marsh systems rather than chronic eutrophication. Such pulses can occur from storm-driven redistribution of organic matter or disease-driven mass mortality events, both of which are increasing globally under climate change. The objective, therefore, was to investigate the transcriptional response of a microbial community provided with chitin rich substrates, namely, crab and lobster shell compost (subsequently referred to as shell compost). A dynamic response of the microbial community was observed within 48 h, characterized by shifts to chitin degradation and stress responses, accompanied by changes in abundance of specific pathogenic taxa.

## Materials and methods

### Site description and sample collection

Soil samples were collected from the Great Marsh, Ipswich MA, USA (42°40′20.5″N 70°48′31.8″W). The sampling area is considered an open marsh, drained by the Labor-in-Vain Creek, which experiences flooding during high tide and drainage during low tide. The site is predominantly high marsh (elevation >1.3 m) with semidiurnal tides of an average mean range of 2.5–2.9 m [37]. The marsh is dominated by saltmeadow cordgrass, *Sporobolus pumilus*, formerly *Spartina patens* [32, 38].

Samples were collected on 2 October 2023 at 7:15 a.m. during low tide. At the time of sampling, the air and soil temperatures were 16°C and 15°C, respectively. Soil pH, determined using VWR pH-Test strips (BDH35309.606), was ~6.0. Soil samples were collected from mud flats, at 0–10 cm depth, adjacent to *S. pumilus* growth, a habitat suitable for chitin-rich crustaceans and mollusks [39]. Collected in bulk, the soil samples were stored in sterile high-density polypropylene bottles at ambient temperature for transport. Samples were immediately processed, about 20 minutes post sample collection.

### Microcosm experiments

The collected soil samples were homogenized and passed through a 2.36 mm sieve to remove larger debris. Two microcosms were prepared, each containing 600 g of homogenized soil, in separate sterile glass containers. Microcosms were kept at room temperature (20°C–22°C, reflective of typical early-October temperatures in northern Massachusetts), sealed but not airtight, and the sediment-maintained moisture saturation for the duration of the study (Fig. S1). One microcosm served as a control, containing only homogenized marsh soil. The second was amended with 10% w/w autoclaved, ground, Neptune’s Harvest crab and lobster shell compost. The total nitrogen and organic carbon content is 5% and 15%–20% respectively, resulting in a C:N of 3:1–4:1. Shell compost was selected rather than purified chitin to reflect more accurately substrates occurring in natural environments. It is also worth noting that purified/chemically derived chitin is less preferred for degradation by microorganisms [40, 41]. The microcosms were mixed using sterile spatulas and sampled at predetermined time points (0, 1, 4, 8, 24, and 48 h), by collecting 2 g aliquots in duplicate. Extracted samples were flash-frozen using an ethanol/dry ice slurry and stored at −80°C until further analysis.

### Preparation of nucleic acid

A total of 24 samples, consisting of control and treatment microcosms recovered from six time points in technical duplicate, were selected for shotgun metagenomic sequencing. Total DNA was prepared from 0.25 g of soil per replicate using DNeasy PowerSoil Pro Kit (Qiagen, Hilden, Germany, 47014). OneStep PCR Inhibitor Removal Kit (Zymo Research Corp, Irvine, CA, USA, D6030) was used to treat and concentrate DNA preparations. Double-stranded DNA concentrations and purity ratios were determined using NanoDrop One Microvolume UV Spectrophotometer (ThermoScientific, Waltham, MA, USA).

RNA for metatranscriptomic sequencing and analysis was extracted and prepared as described. RNeasy® PowerSoil® Total RNA kits (Qiagen, Hilden, Germany, 12866-25) were used to extract total RNA from 2 g of soil for each replicate, following manufacturer’s instructions, with the following modifications. The lysis/bead bashing step was increased to 20 min. During isopropanol precipitation, samples were incubated at −20°C for 45 min. The OneStep PCR Inhibitor Removal Kit (Zymo Research Corp, Irvine, CA, USA, D6030) was used to treat and concentrate purified RNA, following manufacturer’s instructions. RNA concentration and purity were determined using TapeStation 4200 with the RNA Screen Tape (Lexington, MA, USA) and NanoDrop One (ThermoScientific, Waltham, MA, USA). Samples were stored at −80°C until continuation of library preparation.

### Library preparation and sequencing

For each metagenomic sample, 50 ng of purified DNA was sheared to 200 bp using the Covaris S2 Focused Ultrasonicator (Covaris, Woburn, MA, USA). Sequencing libraries were prepared using NEBNext Ultra™ II DNA Library Prep Kit for Illumina (New England Biolabs, Ipswich, MA, USA, E7645). NEBNext Multiplex Oligos for Illumina (New England Biolabs, Ipswich, MA, USA, E6444) were used for indexing and PCR amplification (five cycles). Library concentration and size were assessed using D1000 High Sensitivity Screen Tapes with the TapeStation 4200 (Agilent, Lexington, MA, USA, 5067-5585). DNA libraries were sequenced at the New England Biolabs Sequencing Core, using NovaSeq S2/SP System (Illumina, San Diego, CA, USA) targeting >60 M paired-end reads per sample.

For each metatranscriptomic sample, 800 ng of total RNA was used for library preparation. Ribosomal depletion was completed using NEBNext® rRNA Depletion Kit for Bacteria (New England Biolabs, Ipswich, MA, USA, E7850), following the manufacturer’s instructions. Libraries with ~200 bp inserts were prepared with NEBNext® Ultra II Directional RNA Library Prep Kit (New England Biolabs, Ipswich, MA, USA, E7765). NEBNext® Multiplex Oligos for Illumina® were used for indexing and PCR amplification (six cycles). Library concentration and size were assessed using a TapeStation 4200 with a D1000 High Sensitivity Screen Tape (Lexington, MA, USA). Shotgun metatranscriptomics targeted >100 M paired-end reads per sample.

### Preprocessing of sequencing reads

Quality trimming and adaptor removal from paired read libraries was completed with trimgalore v.0.6.10 [42] under default settings. Trimmed read files were assessed using FastQC v.0.12.1.

### Whole community microbiome profiling

Metagenomic and metatranscriptomic reads were profiled with Kraken2 v.2.1.3 [43] using a confidence score of 0.3, and the Standard Collection database (October 2024). Taxonomic profiles were visualized using Phylosmith v1.0.6 [44] and presented as relative read abundance.

Depth of sequencing read libraries was normalized to the sample with the minimum number of sequenced reads using the “rarefy_even_depth” function of phyloseq v1.46 [45]. Alpha diversity metrics were obtained using the phyloseq “estimate_richness” function. The observed (number of taxa), Shannon (entropy) [46], and inverse Simpson’s diversity index (richness under uniform evenness) [47] were visualized using “plot_richness.” Raw count data were also transformed into relative abundances, expressed as counts per million, and used for principal coordinate analysis (PCoA) employing Bray–Curtis [48, 49] distance measure through phyloseq’s “ordinate” function.

Differential abundance analysis was performed using DESeq2 v1.42.1 [50]. A DESeqDataSet object was generated using the “phyloseq_to_deseq2” function. Taxon-level DESeq2 results are generated from technical duplication, a limitation of these results. Generalized linear models were fitted using control samples as reference by calculating the ratio of their likelihoods. Resulting *P*-values were adjusted using the Benjamini–Hochberg procedure [51] (False Discovery Rate (FDR) ≤ 0.01). Taxa were considered significant if they exhibited differential abundance at late timepoints (24 and 48 h) relative to early timepoints (1 and 4 h). Results are reported as log_2_-fold changes.

### Transcriptional profiling in response to treatment

To assess the effects of shell compost addition on community dynamics, the metaGPA pipeline [52] (https://github.com/linyc74/MetaGPA), which performs phenotype association on the protein domain level, was used. This approach identifies significantly associated protein domains encoded in transcripts based on normalized relative abundance. Performing association analyses on protein domains versus open reading frames provides greater statistical power, and enables tracking of global responses across organisms that drive key functions with differential genes. This approach involves transcript assembly via rnaSPADES v3.15.3 [53], read mapping via bowtie2 v2.5.4 [54], and protein domain annotation using Hidden Markov modelling (HMM). Domains found in transcripts associated within the treatment are considered upregulated, while those associated within the control are considered downregulated. This approach considers all transcripts that encode the same protein domain, making it possible to determine which functions are globally upregulated or downregulated in response to treatment. This analytical pipeline is described in greater detail elsewhere [55].

A variety of gene families containing functional domains of interest were analyzed. Assembled transcripts were annotated using the Protein Family Database, PFAM [56], v.35.0, and HMM was used to annotate via hmmer v.3.3.2 under default settings. PFAM annotations with an *e*-value of ≤1.00 *e*-05 were retained for further analysis. Fisher’s exact test was used to determine if transcripts containing domains were significantly enriched or depleted in the treatment (*P*-value ≤ 1.00 *e*-05), representing upregulated or downregulated functions. Sequences containing domains with significant association scores (*P*-value < 1*e*-20) were extracted and Normalized Transcriptional Changes calculated. First, to correct for sequencing depth and changes in community composition over time, RNA relative abundance was normalized to DNA relative abundance using the following equation:


\begin{align*} &Normalized\ RNA\ Abundance\\&=\frac{RNA\ reads\ mapped\ to\ transcript\ X\div total\ RNA\ reads}{DNA\ reads\ mapped\ to\ transcript\ X\div total\ DNA\ reads} \end{align*}

Next, the ratio between Normalized RNA Abundance in treatment and control for each transcript at each corresponding time point was calculated and log_2_-transformed. This enables tracking of transcriptional changes relative to the control and normalizes changes driven by incubation conditions. The following equation was used to calculate Normalized Transcriptional Changes:


\begin{align*} &Normalized\ Transcriptional\ Changes\\&= {\log}_{2}\frac{Normalized\ RNA\ Abundance\ (Treatment)}{Normalized\ RNA\ Abundance\ (Control)} \end{align*}

### Gene profiling for antimicrobial resistance and virulence determinants

Presence and relative expression of antimicrobial resistance genes and virulence determinants were profiled and quantified using ShortBRED v0.9.3. Custom cluster databases were constructed using the NCBI Pathogen Detection Reference Gene Catalog [57] and Virulence Factor Database [58] with the “-ShortBRED-Identify” function. Sample reads were mapped against the databases using “-ShortBRED-Quantify,” relatively quantified, and reported as reads per kilobase of transcript per million reads mapped (RPKM). Sequences that were present in at least one sample with an RPKM ≥2 or ≥ 1 are displayed in the main text and supplemental, respectively.

### Data visualization

Statistical analysis and figure rendering were completed using R and R Studio v.4.4.2 via the tidyverse collection.

## Results

### Shell compost addition alters species diversity and community composition

Microcosm microbial communities exhibited shifts in taxonomic composition at later time points in samples treated with shell compost compared to untreated samples. Measurements of alpha diversity indicated decreased species diversity in samples collected 24 and 48 h after shell compost addition, observed by inverse Simpson, suggesting changes in evenness and dominance (Fig. 1A). Beta diversity, measured by Bray–Curtis dissimilarity, indicated similar community composition in samples collected before 24 h regardless of treatment, while samples collected at 24 and 48 h were distinct relative to sampling time and treatment (Fig. 1B). Shifts in community composition 24–48 h after shell compost addition were largely driven by a relative increase in Gammaproteobacteria and Bacilli, consistent with previous analyses [55]. An increase in Epsilonproteobacteria was also observed at 48 h in both treatment and untreated control samples and remains an unresolved observation (Fig. 1C). Several bacterial genera represented this compositional shift, including notable increases in *Vibrio*, *Shewanella*, *Aeromonas*, and *Bacillus* spp., with *Acinetobacter* spp. also increasing at 48 h (Fig. 1D and E). In contrast to treated samples, untreated samples remained relatively stable in community composition from 0 to 48 h (Fig. 1).

**Figure 1:**
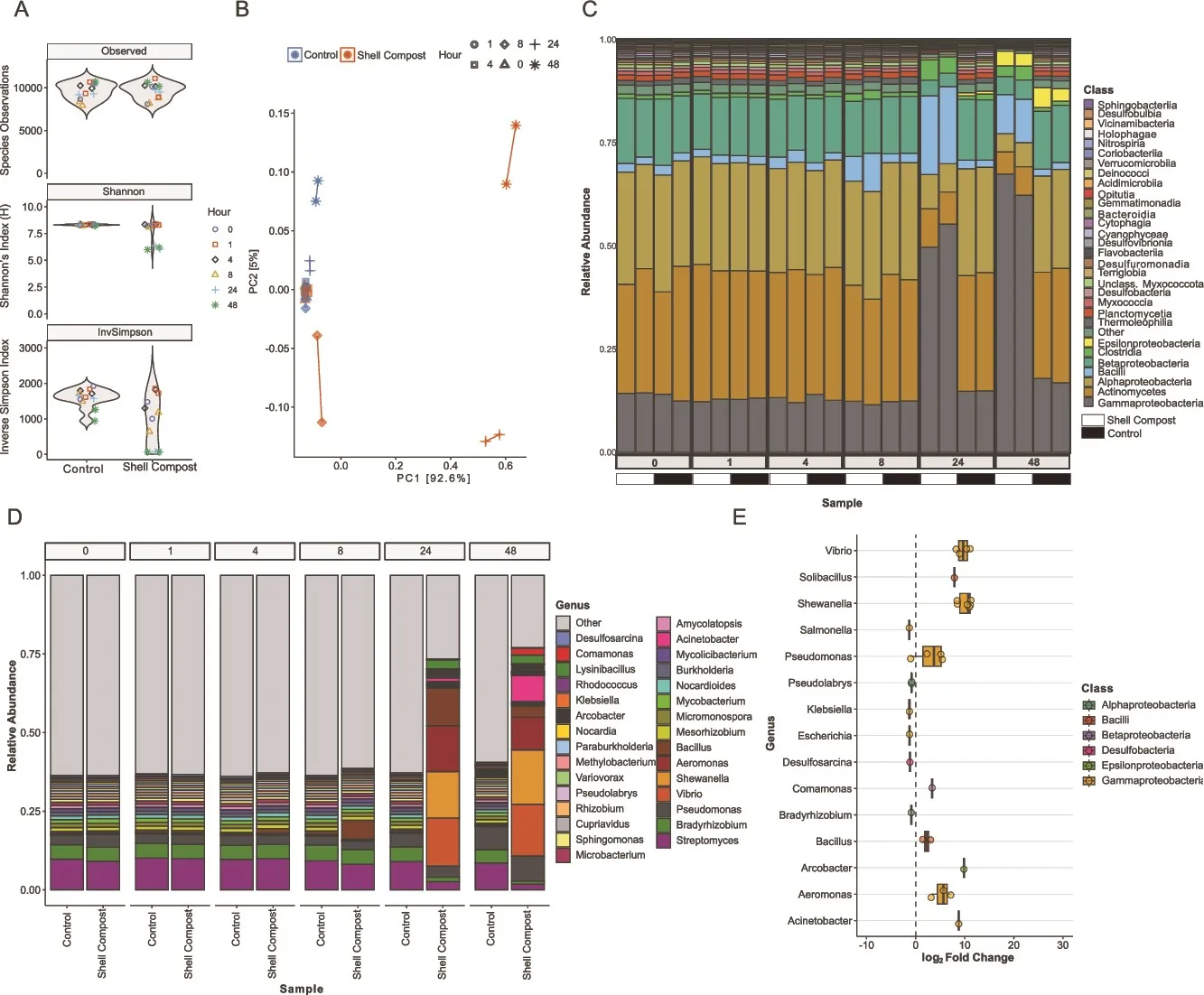
Microbial community composition. (A) Violin plots showing alpha diversity between microbiome taxonomic profiling sets. (B) Principal coordinate analysis of microbial communities. (C) Stacked bar plot showing relative sequencing read abundance of bacterial classes for each replicate sequenced. White and black boxes denote shell compost–treated and control samples, respectively. (D) Stacked bar plot showing average relative sequencing read abundance for bacterial genera. The top 30 most abundant genera are shown, while the rest are denoted “Other” (colored gray). (E) Boxplot showing log_2_ fold change estimates of genera among most abundant genera.

### Compost addition alters the microbial community transcript pool

Metatranscriptomic read profiling exhibited distinct patterns, representing differences in microbial community composition and transcriptional activity. For example, metagenomic and metatranscriptomic read profiling did not exhibit a 1:1 relationship for several taxa, including *Vibrio* and *Aeromonas* spp., which increased in relative abundance but represented only a small portion of total transcripts. Transcriptional alpha diversity was similar among time points regardless of treatment, although indices were lower compared to metagenomic read profiling (Figs 1A and 2A). Beta diversity revealed more similar transcript pools for earlier timepoints (0–8 h), regardless of treatment, with distinct transcript pools at 24–48 h for both control and treated samples (Fig. 2B). Compared to metagenomic read profiling, shifts in the transcript pool were less dynamic, with Gammaproteobacteria, Bacilli, and Clostridia comprising >40% of the profiled transcripts across samples. The genera *Pseudomonas* and *Bacillus* comprised ca. 25% of the transcript pool, regardless of sampling type (Figs 1C and 2C). However, 24 and 48 h after shell compost addition, *Acinetobacter, Lysinibacillus, Clostridium, Aeromonas*, and *Solibacillus* spp. increased in relative transcript abundance. *Arcobacter* spp. and *Comamonas* spp. transcripts increased in control and shell compost–treated samples respectively at 48 h (Fig. 2D and E).

**Figure 2:**
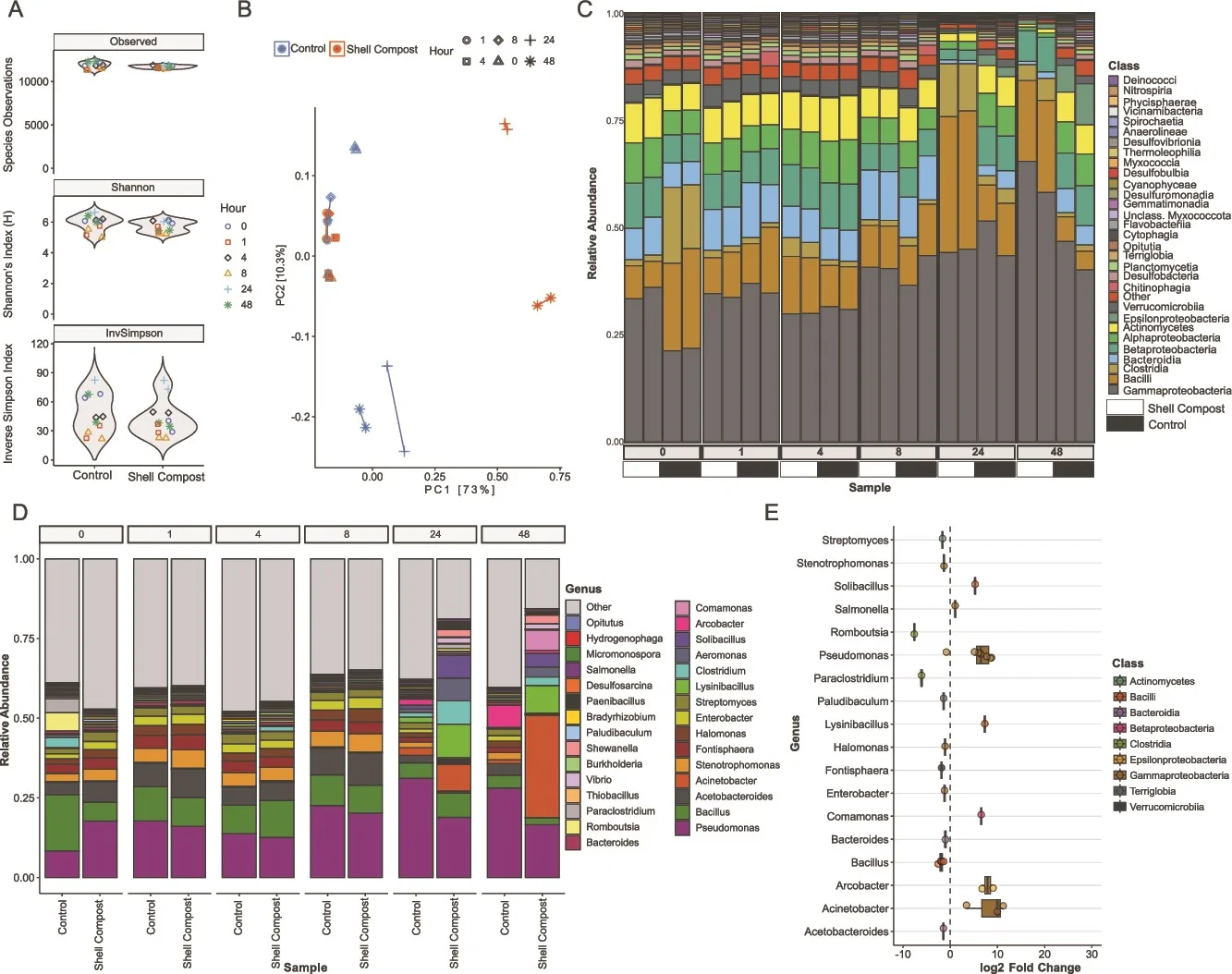
Metatranscript pool composition. (A) Violin plots showing alpha diversity between microbiome transcript profiling sets. (B) Principal coordinate analysis of microbial community transcript pool. (C) Stacked bar plot showing relative transcript read abundance of bacterial classes for each replicate. White and black boxes denote shell compost–treated and control samples, respectively. (D) Stacked bar plot showing averaged relative transcript read abundance for bacterial genera. The top 30 most abundant genera are shown, while the rest are denoted as “Other” (colored gray). (E) Boxplot showing log_2_ fold change estimates of genera among most abundant genera.

To identify metabolically active microbial taxa, transcriptional abundance and taxonomic profiles of both *recA* and *rpoB* genes were analyzed. These widely conserved bacterial housekeeping genes are generally transcriptionally active to maintain core cellular processes [59, 60]. The *recA* gene is essential for homologous recombination and DNA repair [59], while *rpoB* encodes the β subunit of RNA polymerase [61], a proxy for active transcription and metabolic activity. The protein domains responsible for *recA* and *rpoB* activity (PF00154, PF10385) were not enriched or depleted 48 h after shell compost addition (*P*-value > .999), in line with the assumption that protein function is required for basic cellular maintenance and survival. Consistent with metagenomic and metatranscriptomic profiling, *recA* profiling indicated enrichment of Gammaproteobacteria and Bacilli in the treated samples, while a more diverse consortium of bacteria, capable of several metabolisms was observed in the control (Fig. S2, Table S1). It is concluded that the respective bacterial classes in each group were metabolically active members of the treated or control microbial communities.

### Domain enrichment 48 h post shell compost addition

To identify which protein domains, and associated functions, were selected and/or transcriptionally regulated in response to shell compost addition after 48 h, phenotype association analyses were performed (see Materials and Methods). Protein domains served as functional units to include all genes predicted to encode for a similar activity, regardless of taxonomy, enabling assessment of global functional changes throughout the community. An increase at the normalized RNA level indicates transcriptional upregulation of genes harboring this domain across the community. These domains were designated as ‘functionally enriched’ and corresponding transcripts considered ‘upregulated’. Conversely, domains more abundant in control samples were classified as ‘functionally depleted’ in the treated microcosm and their corresponding transcripts designated ‘downregulated’.

Association analyses yielded 108 functionally enriched domains (*P* < 1*e*-20, Table S2). Notably, the glycoside hydrolase family 18 (GH18, PF00704) domain was enriched, the same domain in chitin-degrading enzymes. Other functionally enriched domains included those involved in biosynthesis and metabolism, transport, and protein synthesis. Domains found in proteins involving stress response, resistance, and sporulation were also functionally enriched, suggesting community wide stress response post shell compost addition.

### Chitinase gene regulation

Domains found in enzymes related to chitin degradation were functionally enriched in samples collected 48 h after shell compost addition. Transcripts containing these domains were encoded by a variety of bacterial taxa (Fig. 3A). Most transcripts were identified as containing GH18 domains (EC 3.2.1.14, *n* = 128), previously shown to have endochitinase, exochitinase, and N-acetylglucosaminidase activity [24]. Several upregulated transcripts containing GH18 were assigned to Gammaproteobacteria including *Aeromonas*, *Vibrio*, *Shewanella* spp., and members of the class Bacilli (Fig. 3A). Relative abundance of these taxa increased in soils treated with shell compost after 48 h, contributing to reduced species diversity (Fig. 1). The GH19 domain, with known exochitinase activity [24], was less frequently detected (EC 3.2.1.14, *n* = 19) and assigned to several bacteria, including both *Aeromonas* and *Vibrio* spp. (Figure 3A). Several upregulated transcripts were also identified to encode GH20 domains (EC 3.2.1.52, *n* = 43), annotated with beta-N-acetylglucosaminidase activity, catalyzing breakdown of diacetylchitobiose into N-acetylglucosamine [22].The abundance of transcripts from both identified and unidentified bacterial taxa indicates multiple microbial species contribute to chitin degradation (Fig. 3A).

**Figure 3:**
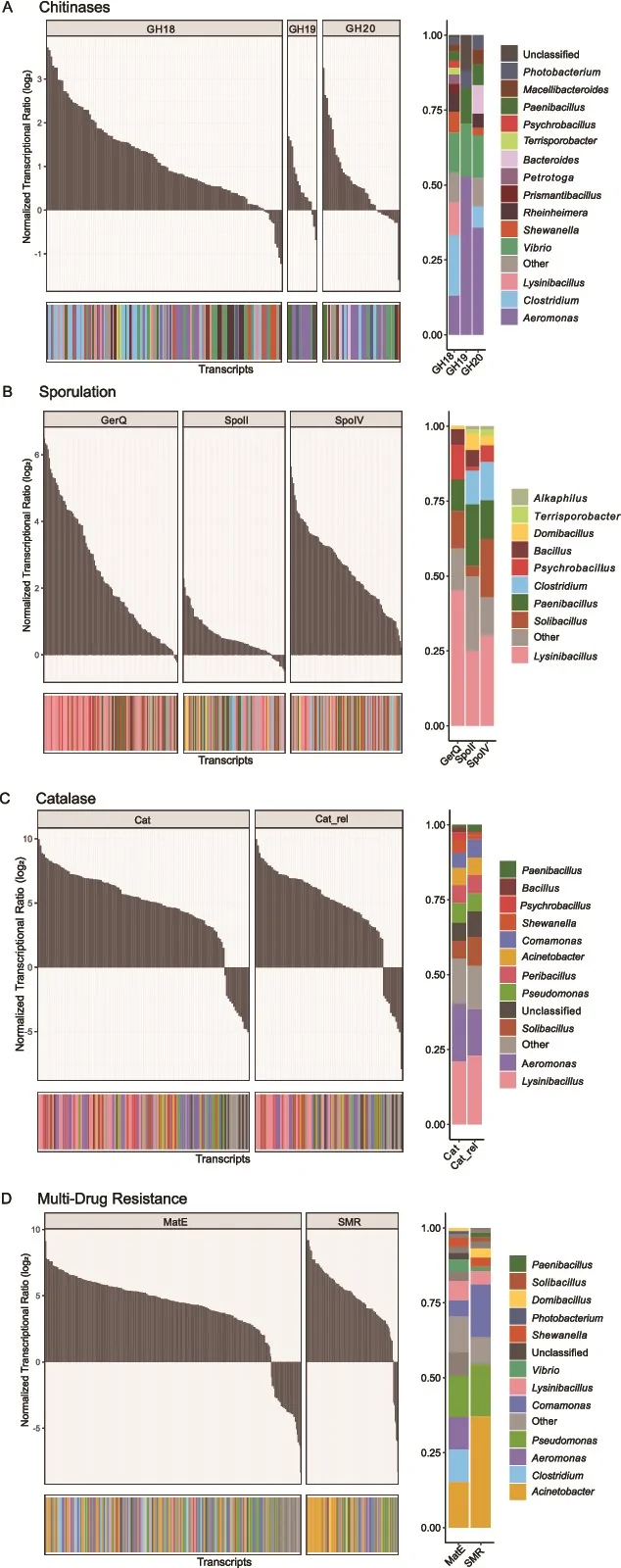
Upregulation community response to shell compost addition. Transcripts encoding select domains with significant enrichment 48 h after chitin addition are depicted with normalized relative transcriptional changes and taxonomic assignment including those with (A) chitinase, (B) sporulation, (C) catalase, and (D) multi-drug resistance annotations. Normalized transcriptional changes are calculated by taking the ratio of relative RNA:DNA from shell compost treated to control samples at 48 h, and DNA ratios = normalized DNA abundance in treatment/normalized DNA abundance in control at 48 h (see Materials and Methods).

### Regulation of genes related to stress

Catalase function (EC 1.11.1.6) was upregulated 48 h after shell compost addition. Functional enrichment was observed for catalase (PF00199) and catalase-related immune-responsive domains (PF06628), both of which are involved in conversion of hydrogen peroxide to water and oxygen under oxidative stress. The most upregulated transcripts containing catalase domains were assigned to *Lysinibacillus* spp. and other Bacilli. However, several transcripts were also assigned to *Aeromonas*, *Pseudomonas*, *Acinetobacter*, and *Comamonas* spp. Despite being catalase positive, *Vibrio* catalase transcripts were not detected (Fig. 3C).

Functional enrichment of domains involved in aspects of sporulation were also observed in samples collected 48 h post shell compost addition. These domains included GerQ (PF09671), a spore coat protein, stage II sporulation protein SpoII (PF08486), involved in earlier steps of sporulation, and putative stage IV sporulation protein YqfD (PF06898), an essential protein for sporulation, the exact function of which is unknown. Most upregulated transcripts containing these domains were assigned to classes Bacilli, and Clostridia (Fig. 3B). These community members are concluded to be nutrient limited and/or stressed, following shell compost amendment and subsequent changes in community structure.

Protein domains involved in multidrug resistance, Small Multidrug Resistance Protein (SMR, PF00893) and MatE (PF01554), were significantly enriched after shell compost addition (Fig. 3D). MatE-containing transcripts were taxonomically diverse, with over 50% of the upregulated transcripts assigned to *Acinetobacter*, *Clostridium*, *Aeromonas*, and *Pseudomonas*. Upregulated SMR transcripts derived mostly from *Acinetobacter*, *Pseudomonas*, and *Comamonas* spp., taxa that not only increased in relative abundance (Fig. 1) but also contributed to the metatranscript pool post shell compost addition (Fig. 2).

### Domain depletion 48 h after shell compost addition

A total of 113 domains were depleted in the shell compost treated samples at 48 h. Several of these domains included various cytochrome, viral, flagellar, and pili proteins. Significant depletion of domains central to primary metabolism pathways was also observed, including those with functional importance in carbon fixation, sulfur oxidation and reduction, nitrogen utilization, and carbon metabolism (Table S3). Consistent with significant loss in species diversity 48 h after shell compost addition, depletion of domains of various metabolic pathways highlights loss in functional diversity.

### Regulation of other metabolism genes

Transcripts containing domains involved in sulfur oxidation (Sox) were downregulated in shell compost samples 48 h, following shell compost addition. SoxZ (PF08770) and SoxY (PF13501) domain containing proteins form critical complexes required for dissimilatory thiosulfate oxidation [62], an important process in sulfur cycling. Compared to the control, transcripts containing these domains were downregulated, suggesting attenuation of this functional pathway, largely driven by a diverse, but poorly characterized group of taxa (Fig. 4A). Similar patterns were observed for transcripts containing AMO domains that have ammonia and/or methane monooxygenase activity (Fig. 4D). Proteins containing AMO (PF02461) and AMOc (subunit C, PF04896) domains play key roles in nitrogen cycling and breakdown of chemically diverse hydrocarbons. Most of these transcripts derived from *Methylobacter* and *Methylomonas* spp., both of which are methanotrophs, indicating amoA is likely pmoA, encoding the alpha subunit of particulate methane monooxygenase.

**Figure 4:**
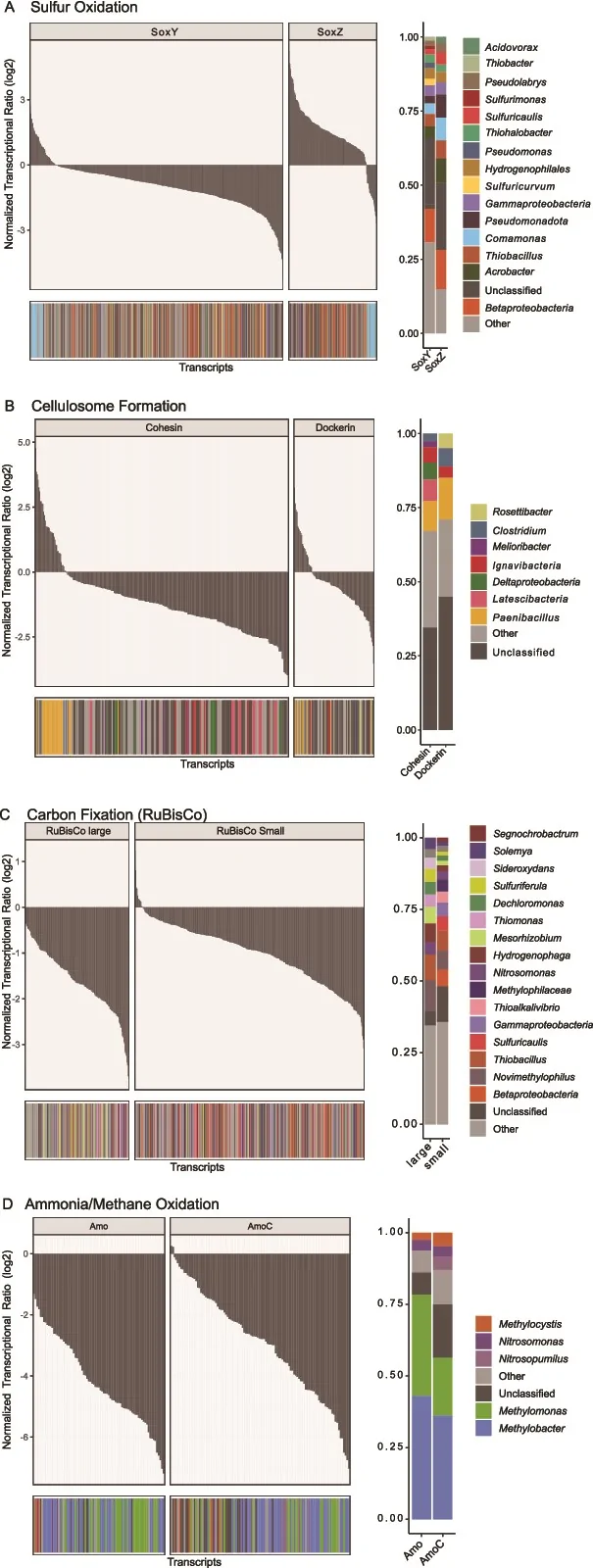
Downregulation community response to shell compost addition. Transcripts encoding select domains with significant depletion 48 h after chitin addition are depicted with normalized relative transcriptional changes and taxonomic assignment including those with (A) sulfur oxidation (B) cellulosome formation (C) carbon fixation (RuBisCO) and (D) ammonia and methane oxidation activity. Normalized transcriptional changes are calculated by taking the ratio of relative RNA:DNA from shell compost treated to control samples at 48 h, and DNA ratios = normalized DNA abundance in treatment/normalized DNA abundance in control at 48 h (see Materials and Methods).

### Regulation of genes related to carbon fixation

Depletion of domains involved in other carbon metabolism pathways was also observed. Transcripts encoding predicted proteins with dockerin (PF00404) and cohesin (PF00963) domains were downregulated 48 h after shell compost addition (Fig. 4B). These proteins are required for the formation of the cellulosome, a multi-enzyme complex essential for cellulose degradation [63]. Similarly, transcripts encoding subunits of ribulose-1,5-bisphosphate carboxylase/oxygenase (RuBisCO) were also downregulated (Fig. 4C). Both large (PF00016) and small (PF00101) subunits were depleted, indicating a functional decrease in carbon fixation.

### Detection of genes related to virulence and antimicrobial resistance

Given several, potentially pathogenic taxa were enriched 24 h after shell compost addition, a targeted analysis was used to confirm their presence and the expression of genes related to virulence and antimicrobial resistance. Several virulence factor (VF) genes were detected at 24 and 48 h (Figs 5 and S3). Their functions varied but included those involved in adherence, effector delivery, and motility. Most VFs detected were from *Aeromonas* and *Vibrio* spp. (Figures 5A and B and S3). Genes encoding exotoxins were detected, including: (i) *aerA* (aerolysin A) and *rtx* (RTX toxins) from *Aeromonas*; (ii) *hlyA* (hemolysin) from *Vibrio* spp.; and (iii) *alo* (anthrolysin O) among other *Bacillus* exotoxin genes. A smaller subset of VFs was found actively expressed 24 and 48 h after shell compost addition (Fig. 6A and B, S3), including those involved in effector delivery from *Acinetobacter*, *Aeromonas*, and *Vibrio* spp. Only one exotoxin gene, *aerA*, showed detectable transcriptional levels (Fig. 6A). Elevated relative transcript abundance of VFs, primarily motility-related genes, were observed at time zero in the shell compost microcosm, an interesting observation as yet unexplained. (Figure 6B).

**Figure 5:**
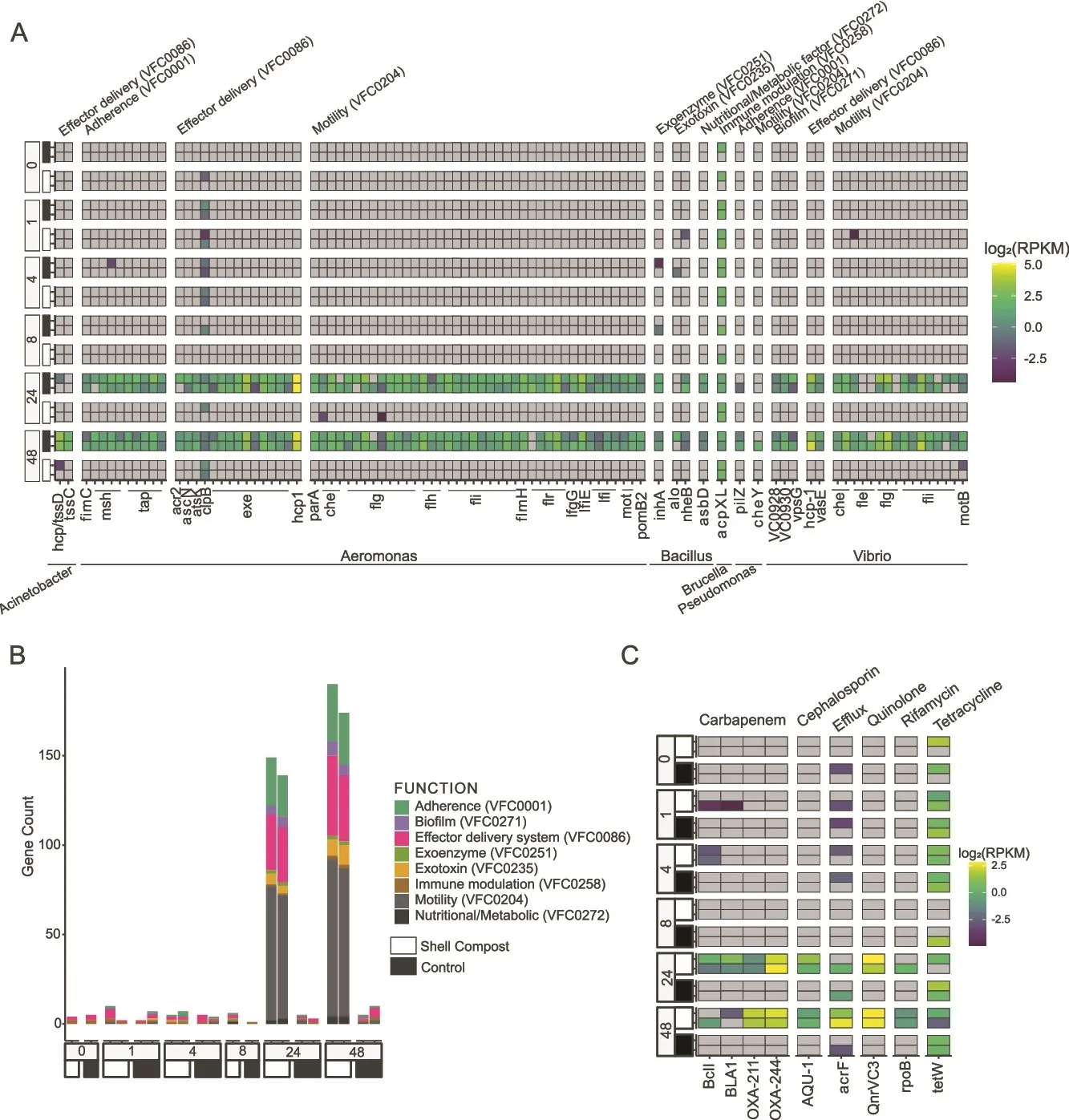
DNA-level relative abundance of virulence factor determinants and antimicrobial resistance. (A) Detection and relative abundance of virulence factor genes where RPKM ≥2 in at least one sample. (B) Stacked bar plots showing gene count for each replicate displayed. (C) Relative gene abundance of detected antimicrobial resistance genes. White and black boxes denote shell compost and control samples, respectively.

**Figure 6:**
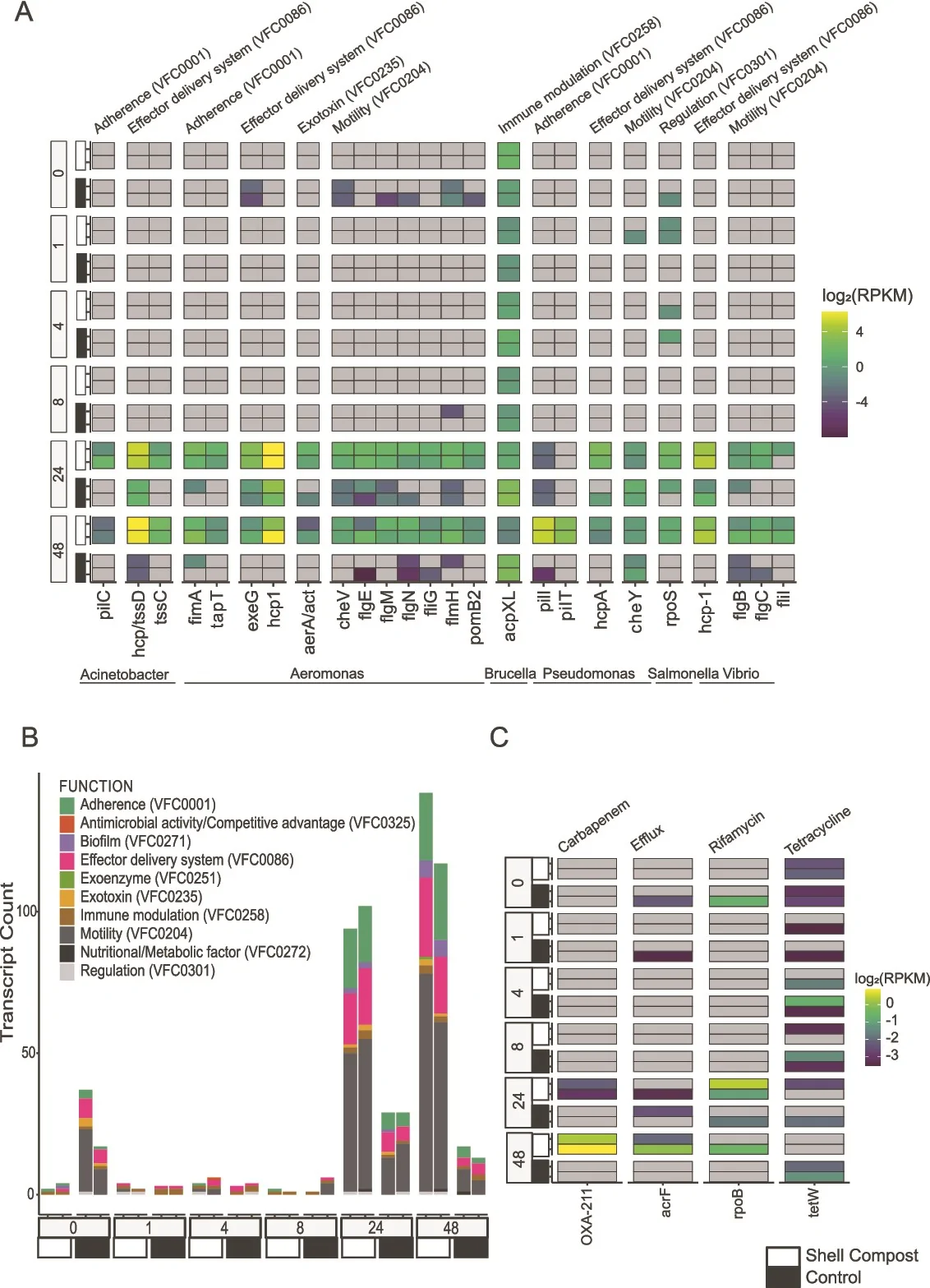
RNA-level relative expression of virulence factor and antimicrobial resistance genes. (A) Detection and relative expression of virulence factor genes where RPKM ≥2 in at least one sample. (B) Stacked bar plots show relative transcript count for each replicate. (C) Relative gene expression of detected antimicrobial resistance genes. White and black boxes denote shell compost treated and control samples, respectively.

Presence and expression of genes involved in antimicrobial resistance (ARGs) were also assessed. Relative abundance of these genes was lower than VF genes (Fig. 5C). Tetracycline resistance gene *tetW* was detected in nearly every sample, regardless of treatment, and exhibited low, but detectable, levels of relative expression (Figs 5C and 6C). At 24 and 48 h post shell compost addition, several genes responsible for carbapenem resistance were detected (Fig. 5C). Expression of ARGs was found to be sporadic, with OXA-211 showing greatest relative expression 48 h after shell compost addition (Fig. 6C). While several ARGs were detected, they were upregulated to a lesser extent than VFs (Figs 5C and 6C).

## Discussion

As the ecosystems of native environments continue to be altered by climatic shifts and nutrient loading, microbial community dynamics need to be investigated to understand their function and resilience. Using a natural, chitin-rich substrate demonstrated how a single pulse event selectively enriches chitin degraders, some of which are potential pathogens autochthonous to coastal ecosystems [64–66]. Dynamic shifts in relative transcript abundance revealed not only an increase in chitin degradation but also stress and virulence responses (Figs 3, 5, and  6). While metagenomic analysis revealed a marked increase in *Aeromonas*, *Vibrio*, and *Shewanella* spp., other bacterial genera showing modest increase in abundance, e.g. *Acinetobacter* and *Clostridium* spp., were some of the most transcriptionally active (Figs 1 and 2). The decoupling between relative abundance and transcriptional activity supports either rapid replication of select taxa showing low transcriptional activity per cell or relative DNA enrichment of surviving taxa.

It was hypothesized that shell compost addition provided a nutrient-rich substrate that would stimulate selected growth and biofilm formation. Ultimately, this resulted in a functional shift that included virulence and resistance factors (Fig. 7). While the hypothesized upregulation of chitin degrading genes was observed, upregulation in other genes related to stress response and resistance were also detected. Species diversity was also reduced, with a loss of functionally important traits driving biogeochemical cycling, evidenced by downregulation of genes such as those involved in carbon fixation, sulfur, and nitrogen metabolism (Figs 1 and 4, Table S3).

**Figure 7:**
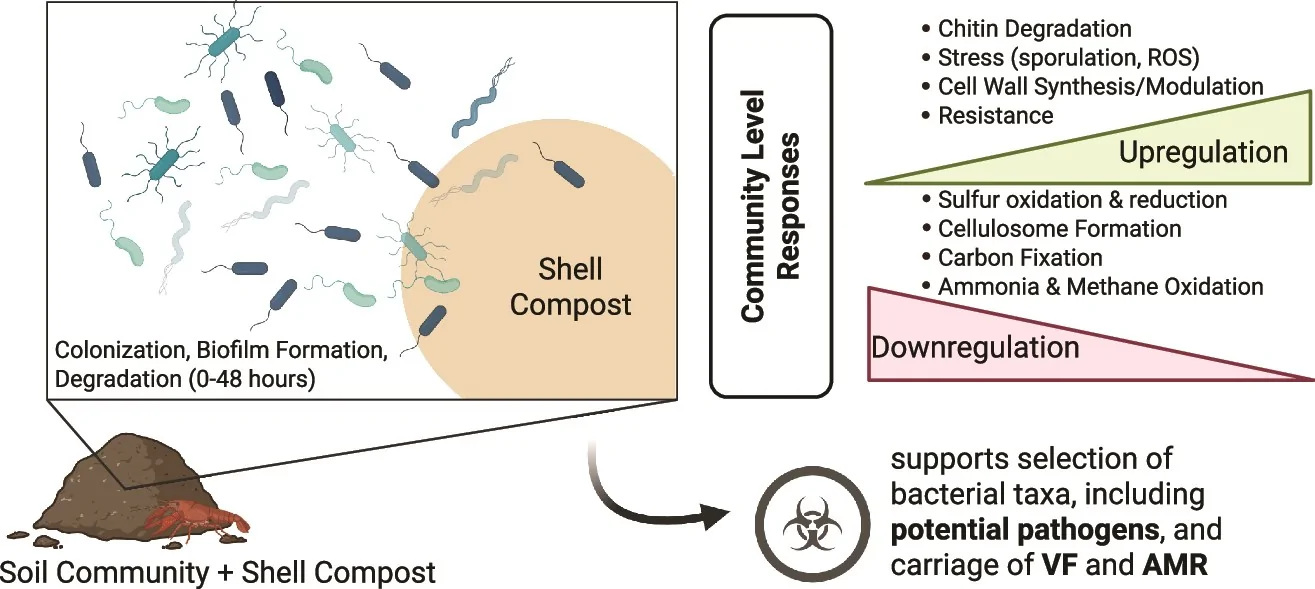
Schematic overview of microbiome response to shell compost addition. Community succession takes place in as little as 48 h, resulting in upregulation and downregulation of functionally important pathways. At 48 h, detection of potentially pathogenic taxa increases, in addition to detection and relative expression of virulence factors (VFs) and antibiotic resistance genes (ARGs), with potential implications for human health. This figure was made with BioRender.

Upregulation of catalase genes across many bacterial taxa suggests community wide response to oxidative stress, reflecting cascading effects of chitin addition. This is supported by greater relative expression of catalase-associated transcripts in treated samples compared to that of the controls, indicating a response driven by amendment rather than differences in oxygen saturation compared to *in situ* conditions. We hypothesize that shell compost acts as a metabolic stimulus, enhancing respiration, increasing metabolic activity, and triggering selective proliferation and competitive interactions [67, 68]. This is consistent with evidence that reactive oxygen species are detected in intertidal sediments containing anoxic zones [69].

Sporulation genes were also upregulated, indicating nutrient limitation and/or stress in Bacillota spp. exposed to unfavorable environmental conditions [70–72]. This response likely represents an alternative survival strategy under highly competitive settings. The upregulation of genes involved in multi-drug resistance that was observed suggests active competition among community members for the chitin-rich substrate. Genes coding for motility, biofilm formation/adherence, and effector delivery were enriched 24–48 h after chitin addition and detectably expressed. A large portion of the VF genes that were detected and expressed were those of *Aeromonas* and *Vibrio* spp., implicating these taxa in potentially virulent responses.

The findings reported here contribute to a growing body of work highlighting the selective growth and reproduction of microbial species, some of which are potentially pathogenic, in northern latitudes under changing climate and nutrient loading scenarios. ARGs were detected in the same amended communities, a growing concern in natural systems worldwide [73]. Warming temperature, changes in salinity and pH, and extreme events such as flooding can trigger selective proliferation of microbial species, including potential pathogens, in coastal ecosystems [17, 21]. Under these conditions, chitin-rich substrates discharging into aquatic ecosystems provide a nutrient source that promotes growth and biofilm formation. Other investigations have demonstrated storm driven nutrient influx into coastal shellfish beds enhance biofilm formation, a factor related to virulence [74–77]. Thus, the introduction of chitin-rich substrates can have important predictive and public health implications for coastal communities.

The experiments carried out in this study included technical replication but did not address relevant biological factors such as seasonality. To overcome these inherent limitations, an analysis pipeline, coopted from genome wide association studies, was implemented to assess significant association of transcripts responding to treatment. The approach implements Fisher’s exact test and normalizes library depths to identify significant differences in relative transcript abundance between control and treated microcosms. However, it should be noted that observed changes in community composition cannot be fully distinguished from incubation artifacts without microcosm replicates. Another limitation is that the methods used here measure relative sequencing read abundance and do not permit quantification of absolute changes in bacterial taxa. While results reported here relied on technical replication, and normalization to a control microcosm to account for incubation artifacts, biological replication will allow further determination of the generality of the findings.

An important point to consider based on the results of this study is whether the resilience of coastal communities will withstand significant loss of microbial species diversity and concurrent downregulation of metabolic genes that drive biogeochemical cycles (Figs 1 and 4, Table S3). These results also highlight the potential for certain bacterial taxa, which naturally occur in low abundance, to rapidly dominate the microbial community in response to chitin-rich substrates. Additionally, samples were collected mid-October in northern Massachusetts, a time and location not yet associated with vibriosis and other pathogen outbreaks [64, 78]. As changes in weather patterns and extreme events intensify, in addition to nutrient loading, microbial surveillance of coastal systems will need to be instituted to monitor microbiome stability in providing biogeochemical cycling and associated ecosystem services.

## Supplementary Material

SI_ChitinMultiOmics_August26_ycag307

## Data Availability

Raw reads are available on the NCBI Sequencing Read Archive (SRA) under BioProject: PRJNA1293438.
