# Supplementary material for: Microbial community multi-omic analysis of marsh sediment following enrichment with crustacean shell compost
Source: ISME Commun. 2026 Oct 24;6(1):ycag307. doi: 10.1093/ismeco/ycag307 (PMC13768748; doi:10.1093/ismeco/ycag307)
Supplement: Supplementary file 1 [file si_chitinmultiomics_august26_ycag307.pdf]

Supplemental Figures and Tables

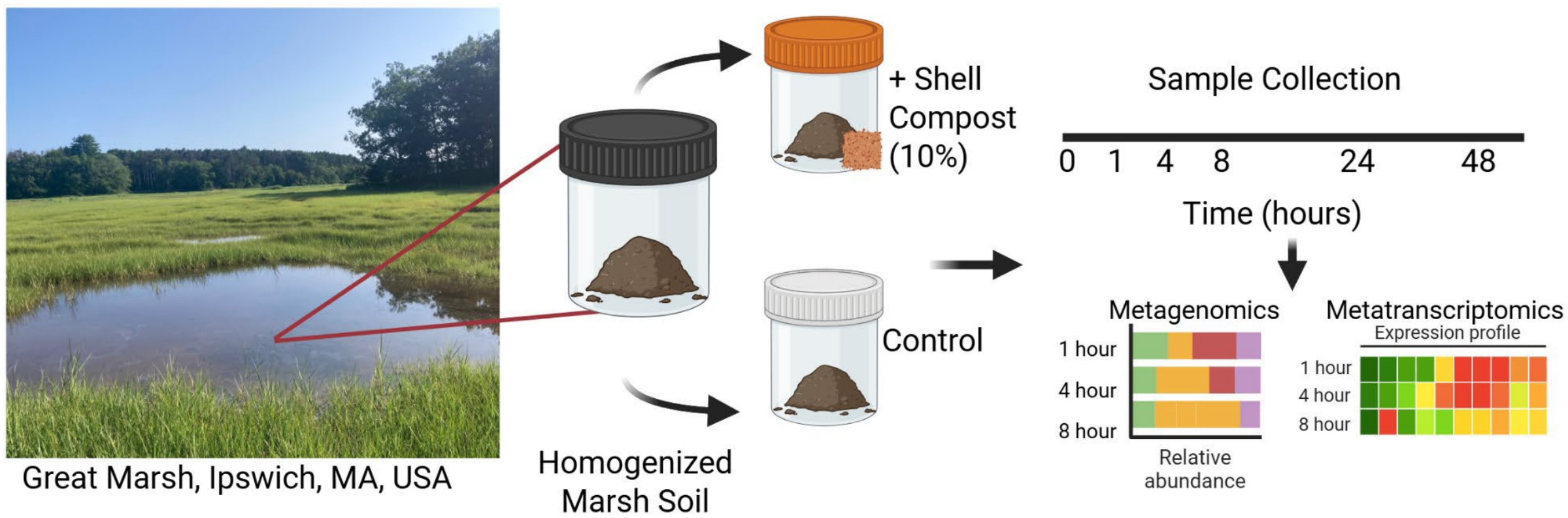

Figure S1: Schematic overview of the microcosm experiment and sample collection. Figure generation was completed with BioRender.

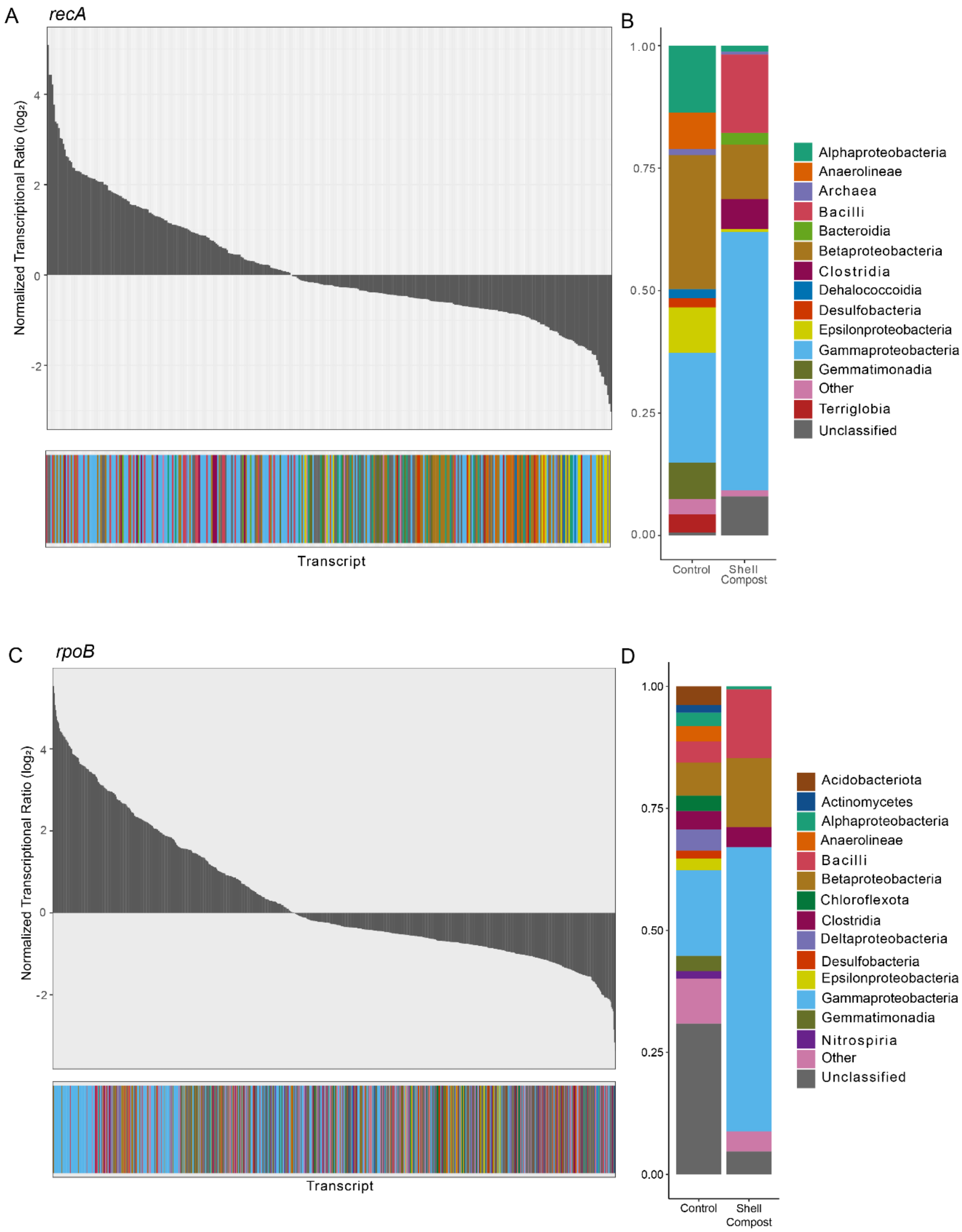

Figure S2: Metatranscriptomic profiling of the recA and rpoB genes. Normalized transcriptional changes for A) recA and C) rpoB 48 hours after chitin addition. B,D) Relative transcript abundance of bacterial classes in the control and treated microcosms for each gene.

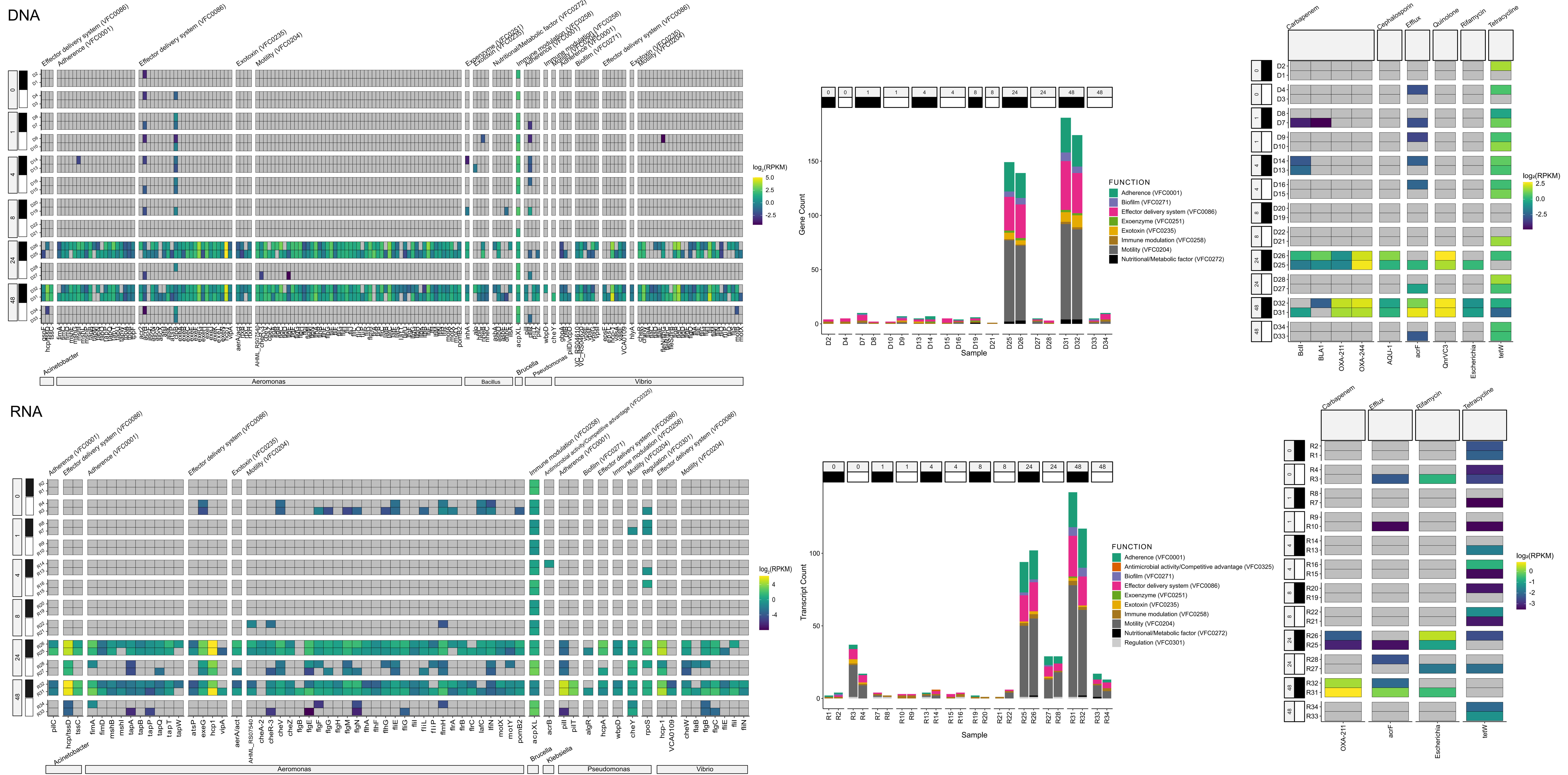
DNA
RNA
Acinetobacter
Aeromonas
Bacillus
Brucella
Pseudomonas
Vibrio
Klebsiella
log2(RPKM)
Gene Count
Transcript Count
Sample
FUNCTION
Adherence (VFC0001)
Antimicrobial activity/Competitive advantage (VFC0325)
Biofilm (VFC0271)
Effector delivery system (VFC0086)
Exoenzyme (VFC0251)
Exotoxin (VFC0235)
Immune modulation (VFC0258)
Motility (VFC0204)
Nutritional/Metabolic factor (VFC0272)
Regulation (VFC0301)
Carbapenem
Cephalosporin
Efflux
Quinolone
Rifamycin
Tetracycline
BcII
BLA1
OXA-211
OXA-244
AQU-1
acrF
QnrVC3
Escherichia
tetW

Figure S3: Microbiome profiling for virulence factor determinants and antimicrobial resistance. A) Detection and relative abundance of virulence factor genes where RPKM ≥1 in at least one sample. Stacked bar plots showing gene count for each replicate are also displayed. B) Detection and relative expression of virulence factor genes. Stacked bar plots showing transcript count for each replicate are also displayed. C) Relative gene abundance and expression of detected antimicrobial resistance genes.

Table S1: Summary of transcripts containing the recA gene. The Node ID represents the transcript identifier from the metatranscriptome assembly. Taxonomy at the Genus and Class level are listed, where assignment was possible. The Normalized Transcriptional Change (See Methods) is shown. Positive and negative transcriptional change indicate greater relative abundance in the shell compost treated or control sample, respectively.

Node ID	Transcript Length (bp)	Transcript Origin	Genus	Class	$\log_2$(Normalized Transcriptional Change)
223450	660	shell compost	Acidovorax	Betaproteobacteria	3.014626336
87489	1326	shell compost	Acinetobacter	Gammaproteobacteria	0.876773265
38687	2157	shell compost	Acinetobacter	Gammaproteobacteria	0.447121122
78893	1415	shell compost	Acinetobacter	Gammaproteobacteria	0.445914797
66568	1501	control	Acinetobacter	Gammaproteobacteria	-1.62051285
23116	2817	shell compost	Acinetobacter	Gammaproteobacteria	0.299975384
57826	1716	shell compost	Acinetobacter	Gammaproteobacteria	0.870245538
64184	1609	shell compost	Acinetobacter	Gammaproteobacteria	0.312922975
92549	1277	shell compost	Acinetobacter	Gammaproteobacteria	1.815844393
111741	1121	shell compost	Acinetobacter	Gammaproteobacteria	4.21441348
10534	4040	shell compost	Acinetobacter	Gammaproteobacteria	0.942811004

100256	1210	shell compost	Acinetobacter	Gammaproteobacteria	2.604772756
71378	1506	shell compost	Acinetobacter	Gammaproteobacteria	1.868811068
112336	1117	shell compost	Acinetobacter	Gammaproteobacteria	0.756678164
10103	4112	shell compost	Acinetobacter	Gammaproteobacteria	1.204632385
44463	1999	shell compost	Acinetobacter	Gammaproteobacteria	1.347333392
25109	2704	shell compost	Acinetobacter	Gammaproteobacteria	1.107124997
28358	2545	shell compost	Acinetobacter	Gammaproteobacteria	2.072291035
4806	5568	shell compost	Acinetobacter	Gammaproteobacteria	1.257337612
62783	1632	shell compost	Acinetobacter	Gammaproteobacteria	3.35106723
47050	1936	shell compost	Acinetobacter	Gammaproteobacteria	1.054254549
46242	1955	shell compost	Acinetobacter	Gammaproteobacteria	1.156206403
19829	3028	shell compost	Acinetobacter	Gammaproteobacteria	1.444643597
140762	1061	control	Acinetobacter	Gammaproteobacteria	2.233468535
140762	1061	control	Acinetobacter	Gammaproteobacteria	0.111222966
14535	3501	shell compost	Acinetobacter	Gammaproteobacteria	1.519338989

15815	3377	shell compost	Acinetobacter	Gammaproteobacteria	1.280750696
15367	3418	shell compost	Acinetobacter	Gammaproteobacteria	1.298055649
8176	4488	shell compost	Acinetobacter	Gammaproteobacteria	2.227754457
27193	2599	shell compost	Acinetobacter	Gammaproteobacteria	1.546049353
173471	955	control	Acrobacter	Epsilonproteobacteria	-1.992950089
16820	2652	control	Acrobacter	Epsilonproteobacteria	-0.710977519
119190	1149	control	Acrobacter	Epsilonproteobacteria	-1.566848308
217115	851	control	Acrobacter	Epsilonproteobacteria	-1.094528728
126067	1030	shell compost	Acrobacter	Epsilonproteobacteria	-3.025346125
62236	1641	shell compost	Aeromonas	Gammaproteobacteria	-0.173679719
56833	1734	shell compost	Aeromonas	Gammaproteobacteria	0.123729863
56830	1734	shell compost	Aeromonas	Gammaproteobacteria	0.067903687

53489	1799	shell compost	Aeromonas	Gammaproteobacteria	0.777063501
56831	1734	shell compost	Aeromonas	Gammaproteobacteria	0.225241462
649	12006	shell compost	Aeromonas	Gammaproteobacteria	0.086000494
14151	3543	shell compost	Aeromonas	Gammaproteobacteria	0.1408082
2082	7746	shell compost	Aeromonas	Gammaproteobacteria	0.398112119
9820	3289	control	Aliarcobacter	Epsilonproteobacteria	-1.567888522
22543	2358	control	Aliarcobacter	Epsilonproteobacteria	-1.369888584
221778	842	control	Aliarcobacter	Epsilonproteobacteria	-1.144409962
77582	1402	control	Aliarcobacter	Epsilonproteobacteria	-2.24459103
439139	586	control	Aliarcobacter	Epsilonproteobacteria	-0.251291907
11100	3131	control	Aliarcobacter	Epsilonproteobacteria	-1.427765873
71180	1457	control	Aliarcobacter	Epsilonproteobacteria	-2.654138676

139749	1064	control	Aliarcobacter	Epsilonproteobacteria	-0.221362631
91795	1299	control	Aliarcobacter	Epsilonproteobacteria	-1.418163271
68023	1487	control	Aliarcobacter	Epsilonproteobacteria	-2.445518831
202106	715	shell compost	Aliarcobacter	Epsilonproteobacteria	-0.141356243
221802	664	shell compost	Alkaliphilus	Clostridia	0.667048605
50691	1690	control	Anaerolinea	Anaerolineae	-0.935834895
152900	1018	control	Aquabacterium	Betaproteobacteria	-0.430533604
144728	1046	control	Azonexus	Betaproteobacteria	-0.986731407
225936	834	control	Azonexus	Betaproteobacteria	-1.329250436
19018	2525	control	Azonexus	Betaproteobacteria	-0.526870935
30892	2432	shell compost	Bacteroides	Bacteroidia	0.311751711

756074	438	control	Bradyrhizobium	Alphaproteobacteria	-0.614806021
17537	3208	shell compost	Brevundimonas	Alphaproteobacteria	1.090937506
22354	2863	shell compost	Brevundimonas	Alphaproteobacteria	1.146024633
63332	1534	control	Burkholderiaceae_bacterium	Betaproteobacteria	0.228096512
57744	1596	control	Burkholderiales_bacterium	Betaproteobacteria	-1.225174896
67291	1494	control	Burkholderiales_bacterium	Betaproteobacteria	-0.769476817
68638	1481	control	Burkholderiales_bacterium	Betaproteobacteria	-0.837726081
206756	873	control	Burkholderiales_bacterium	Betaproteobacteria	-2.249120724
243021	803	control	Burkholderiales_bacterium	Betaproteobacteria	-0.597483431
159746	996	control	Burkholderiales_bacterium	Betaproteobacteria	-0.655343703
179290	939	control	Burkholderiales_bacterium	Betaproteobacteria	-0.288167999

493930	550	control	Burkholderiales_bacterium	Betaproteobacteria	-1.050583308
180836	935	control	Burkholderiales_bacterium	Betaproteobacteria	-1.089945071
190707	910	control	Burkholderiales_bacterium	Betaproteobacteria	-0.800575613
66482	1502	control	Burkholderiales_bacterium	Betaproteobacteria	-0.613441412
228034	830	control	Burkholderiales_bacterium	Betaproteobacteria	-0.45644726
95199	1277	control	Burkholderiales_bacterium	Betaproteobacteria	-0.534496968
133834	1087	control	Burkholderiales_bacterium	Betaproteobacteria	-0.279454592
488993	553	control	Burkholderiales_bacterium	Betaproteobacteria	-0.800490942
84997	1345	control	Burkholderiales_bacterium	Betaproteobacteria	-0.584423133
190705	910	control	Burkholderiales_bacterium	Betaproteobacteria	-0.728333839
113346	1177	control	Burkholderiales_bacterium	Betaproteobacteria	-0.370795393

85301	1343	control	Burkholderiales_bacterium	Betaproteobacteria	-0.387826538
97461	1263	control	Burkholderiales_bacterium	Betaproteobacteria	-0.610107934
335394	478	shell compost	Burkholderiales_bacterium	Betaproteobacteria	0.152714986
391686	623	control	Burkholderiales_bacterium	Betaproteobacteria	-0.599384071
159755	996	control	Burkholderiales_bacterium	Betaproteobacteria	0.31763872
244445	614	shell compost	Burkholderiales_bacterium	Betaproteobacteria	0.264533411
67429	1561	shell compost	Burkholderiales_bacterium	Betaproteobacteria	2.067231829
24362	2285	control	Candidatus_Deferrimicrobium	Other	-0.218961944
92635	1294	control	Candidatus_Deferrimicrobium	Other	-0.154972062
103386	1185	shell compost	Candidatus_Niameybacter	Clostridia	0.878866014
142823	1053	control	Candidatus_Thiodiazotropha	Gammaproteobacteria	0.111222966
328767	485	shell compost	Clostridium	Clostridia	0.3337984
253640	596	shell compost	Clostridium	Clostridia	0.728587994

11855	3826	shell compost	Clostridium	Clostridia	1.701932902
419264	403	shell compost	Clostridium	Clostridia	1.516131801
106027	1164	shell compost	Clostridium	Clostridia	1.28563198
30051	2466	shell compost	Clostridium	Clostridia	2.631341942
76045	1448	shell compost	Comamonadaceae_bacterium	Betaproteobacteria	1.985525036
108589	1144	shell compost	Comamonas	Betaproteobacteria	1.550669144
111612	1122	shell compost	Comamonas	Betaproteobacteria	0.623761439
112449	1116	shell compost	Comamonas	Betaproteobacteria	1.057263516
135337	977	shell compost	Comamonas	Betaproteobacteria	2.376779631
163535	846	shell compost	Comamonas	Betaproteobacteria	1.325139984
422215	401	shell compost	Comamonas	Betaproteobacteria	2.058631482
203796	710	shell compost	Comamonas	Betaproteobacteria	0.75503769
4108	5928	shell compost	Comamonas	Betaproteobacteria	1.074523575
124840	1037	shell compost	Comamonas	Betaproteobacteria	0.948928516
216049	678	shell compost	Comamonas	Betaproteobacteria	3.251648642

83534	1366	shell compost	Comamonas	Betaproteobacteria	3.39179914
68312	1548	shell compost	Comamonas	Betaproteobacteria	2.152071926
81704	1385	shell compost	Comamonas	Betaproteobacteria	1.747011602
112439	1116	shell compost	Comamonas	Betaproteobacteria	2.299900765
443724	387	shell compost	Comamonas	Betaproteobacteria	2.777912663
144726	1046	control	Dechloromonas	Betaproteobacteria	-1.246144872
117284	1158	control	Dechloromonas	Betaproteobacteria	-2.148547146
69803	1470	control	Desulfobacterales_bacterium	Desulfobacteria	-0.703890753
19284	2511	control	Desulfobacterales_bacterium	Desulfobacteria	-0.524960202
19287	2511	control	Desulfobacterales_bacterium	Desulfobacteria	-0.521973825
147293	916	shell compost	Domibacillus	Bacilli	0.91231464
1848	8088	shell compost	Domibacillus	Bacilli	2.21192434
3326	6487	shell compost	Domibacillus	Bacilli	2.310632716

120819	1061	shell compost	Domibacillus	Bacilli	2.534317639
19018	2525	control	Ferribacterium	Betaproteobacteria	-0.526870935
65420	1513	control	Gallionella	Betaproteobacteria	-0.854202773
234494	818	control	Gallionella	Betaproteobacteria	-0.884623794
360833	651	control	Gallionella	Betaproteobacteria	-0.585623135
35504	1963	control	Gallionellaceae_bacterium	Betaproteobacteria	-1.001595266
72085	1449	control	Gemmatimonadaceae_bacterium	Gemmatimonadia	-0.582965087
83904	1353	control	Gemmatimonadaceae_bacterium	Gemmatimonadia	-0.408525263
120135	1145	control	Gemmatimonadaceae_bacterium	Gemmatimonadia	-0.46503858
140680	949	shell compost	Gemmatimonadaceae_bacterium	Gemmatimonadia	0.101399022
117084	1159	control	Gemmatimonadaceae_bacterium	Gemmatimonadia	-0.398109487

109503	1137	shell compost	Lysinibacillus	Bacilli	0.889387164
69457	1532	shell compost	Lysinibacillus	Bacilli	0.448389182
40991	2092	shell compost	Lysinibacillus	Bacilli	0.23722645
173766	807	shell compost	Lysinibacillus	Bacilli	0.588908245
306295	513	shell compost	Lysinibacillus	Bacilli	0.822356527
101527	1200	shell compost	Lysinibacillus	Bacilli	1.026554418
112146	1118	shell compost	Lysinibacillus	Bacilli	1.542232172
114795	1100	shell compost	Lysinibacillus	Bacilli	1.030834954
114794	1100	shell compost	Lysinibacillus	Bacilli	1.111870575
112864	1113	shell compost	Lysinibacillus	Bacilli	1.843411855
113732	1107	shell compost	Lysinibacillus	Bacilli	1.5630184
39426	2136	shell compost	Macellibacteroides	Bacteroidia	0.345833558
144204	931	shell compost	Macellibacteroides	Bacteroidia	1.684830012
14005	3559	shell compost	Macellibacteroides	Bacteroidia	1.493732597
16706	2659	control	Marinobacterium	Gammaproteobacteria	-1.50170116

102352	1234	control	Marinobacterium	Gammaproteobacteria	-1.161127463
123037	1048	shell compost	Marinobacterium	Gammaproteobacteria	2.129071347
72668	1491	shell compost	Methanolinea	Archaea	1.007585122
123427	1130	control	Micropepsaceae_bacterium	Alphaproteobacteria	-1.463438758
107319	1207	control	Micropepsaceae_bacterium	Alphaproteobacteria	-1.44087039
170045	821	shell compost	Myroides	Other	1.211322902
155832	878	shell compost	Oceanirhabdus	Clostridia	0.695656262
4334	5799	shell compost	Oceanisphaera	Gammaproteobacteria	0.842824956
2958	6808	shell compost	Oceanisphaeria	Gammaproteobacteria	1.116527456
28106	2156	control	Oceanobacter	Gammaproteobacteria	-1.589469816
30999	2426	shell compost	Other	Other	1.299060321
140081	952	shell compost	Paenibacillus	Bacilli	1.772676691
188151	757	shell compost	Paenibacillus	Bacilli	2.297079352
109899	1134	shell compost	Paenibacillus	Bacilli	5.087813275

123171	1047	shell compost	Paenibacillus	Bacilli	1.460121872
205267	706	shell compost	Peribacillus	Bacilli	3.764880548
1357122	324	control	Pseudolabrys	Alphaproteobacteria	-0.268759477
336742	676	control	Pseudolabrys	Alphaproteobacteria	-0.384380985
942825	390	control	Pseudolabrys	Alphaproteobacteria	-0.913547846
307567	709	control	Pseudolabrys	Alphaproteobacteria	-0.284954076
412487	606	control	Pseudolabrys	Alphaproteobacteria	-0.208134877
497430	548	control	Pseudolabrys	Alphaproteobacteria	-0.271719834
4	39977	control	Pseudomonas	Gammaproteobacteria	-1.249958566
164439	843	shell compost	Pseudomonas	Gammaproteobacteria	-1.564969761
76606	1410	control	Pseudomonas	Gammaproteobacteria	-1.668435225

6846	3781	control	Pseudomonas	Gammaproteobacteria	-1.655651072
4158	4627	control	Pseudomonas	Gammaproteobacteria	-2.423250135
5262	4213	control	Pseudomonas	Gammaproteobacteria	-1.677368306
7308	4695	shell compost	Pseudomonas	Gammaproteobacteria	-1.766246314
88465	1316	shell compost	Pseudomonas	Gammaproteobacteria	-0.818940482
74297	1469	shell compost	Pseudomonas	Gammaproteobacteria	0.279177668
67950	1554	shell compost	Pseudomonas	Gammaproteobacteria	1.377633602
67950	1554	shell compost	Pseudomonas	Gammaproteobacteria	1.377633602
63381	1622	shell compost	Pseudomonas	Gammaproteobacteria	0.626120836
114799	1100	shell compost	Pseudomonas	Gammaproteobacteria	0.47405404
136954	968	shell compost	Pseudomonas	Gammaproteobacteria	0.452315095
128081	1018	shell compost	Pseudomonas	Gammaproteobacteria	1.75116696
135302	977	shell compost	Pseudomonas	Gammaproteobacteria	2.206869249

141772	943	shell compost	Pseudomonas	Gammaproteobacteria	2.094238817
132363	993	shell compost	Pseudomonas	Gammaproteobacteria	0.48898876
3732	4826	control	Pseudomonas	Gammaproteobacteria	1.138821792
57421	1723	shell compost	Pseudomonas	Gammaproteobacteria	1.875777781
90525	1296	shell compost	Pseudomonas	Gammaproteobacteria	-0.128085104
16964	3262	shell compost	Pseudomonas	Gammaproteobacteria	-0.001472939
50579	1858	shell compost	Pseudomonas	Gammaproteobacteria	3.035105789
182262	777	shell compost	Pseudomonas	Gammaproteobacteria	2.133124412
50552	1859	shell compost	Pseudomonas	Gammaproteobacteria	1.246216227
11444	3890	shell compost	Pseudomonas	Gammaproteobacteria	0.871227167
101125	1203	shell compost	Pseudomonas	Gammaproteobacteria	1.624691661
31217	2418	shell compost	Pseudomonas	Gammaproteobacteria	0.314968947
14310	3525	shell compost	Pseudomonas	Gammaproteobacteria	1.804454857
97984	1229	shell compost	Pseudomonas	Gammaproteobacteria	1.792061531

16272	2686	control	Gemmatimonidales_bacterium	Gemmatimonadia	-0.162140192
16272	2686	control	Gemmatimonidales_bacterium	Gemmatimonadia	-0.162140192
8248	3529	control	Gemmatimonidales_bacterium	Gemmatimonadia	-0.217997627
8248	3529	control	Gemmatimonidales_bacterium	Gemmatimonadia	-0.217997627
7706	3617	control	Geopseudomonas	Gammaproteobacteria	-1.766374032
51420	1841	shell compost	Geopseudomonas	Gammaproteobacteria	-0.221592327
52347	1821	shell compost	Geopseudomonas	Gammaproteobacteria	0.977364504
81129	1374	control	Hydrogenophage	Betaproteobacteria	-0.587833523
135187	978	shell compost	Lysinibacillus	Bacilli	-0.659241198
31121	2422	shell compost	Lysinibacillus	Bacilli	-0.035274598
125895	1031	shell compost	Lysinibacillus	Bacilli	0.590499565

56583	1739	shell compost	Pseudomonas	Gammaproteobacteria	1.485361371
104085	1179	shell compost	Pseudomonas	Gammaproteobacteria	1.462617246
33327	2334	shell compost	Pseudomonas	Gammaproteobacteria	1.081456821
68889	1540	shell compost	Rheinheimera	Gammaproteobacteria	0.085626686
85087	1350	shell compost	Shewanella	Gammaproteobacteria	-0.096959609
3146	6644	shell compost	Shewanella	Gammaproteobacteria	-0.069582626
67710	1557	shell compost	Shewanella	Gammaproteobacteria	0.210692555
49478	1881	shell compost	Shewanella	Gammaproteobacteria	0.223923006
232266	640	shell compost	Solibacillus	Bacilli	0.166141256
83357	1368	shell compost	Solibacillus	Bacilli	0.051537516
79451	1409	shell compost	Solibacillus	Bacilli	0.138354296
118992	1150	control	Stellaceae_bacterium	Alphaproteobacteria	-0.464071225
186092	922	control	Steroidobacteraceae_bacterium	Gammaproteobacteria	-0.380210164

17047	3254	shell compost	Stutzerimonas	Gammaproteobacteria	2.137799049
23608	2785	shell compost	Stutzerimonas	Gammaproteobacteria	1.63834797
28779	2525	shell compost	Stutzerimonas	Gammaproteobacteria	2.063037738
18524	3123	shell compost	Stutzerimonas	Gammaproteobacteria	2.496601351
17264	3233	shell compost	Stutzerimonas	Gammaproteobacteria	2.363361467
3973	6018	shell compost	Stutzerimonas	Gammaproteobacteria	2.16635483
26420	2637	shell compost	Stutzerimonas	Gammaproteobacteria	2.907363833
80854	1376	control	Sulfuricella	Betaproteobacteria	-0.423958475
172593	958	control	Sulfurimicrobium	Betaproteobacteria	-0.568616621
144784	1046	control	Thiobacillaceae_bacterium	Betaproteobacteria	-0.520090432
182785	930	control	Unclassified	Archaea	-1.280173903
131843	1095	control	Unclassified	Unclassified	-1.425575301
28582	2142	control	Unclassified	Archaea	-2.85434149

32705	2030	control	Unclassified	Terriglobia	-1.574815054
188361	916	control	Unclassified	Betaproteobacteria	-0.601871624
138146	1070	control	Unclassified	Unclassified	-0.860638483
38312	1904	control	Unclassified	Gammaproteobacteria	-0.801409231
99958	1248	control	Unclassified	Gammaproteobacteria	-0.744035362
114628	1171	control	Unclassified	Unclassified	-0.93315908
1033835	372	control	Unclassified	Gammaproteobacteria	-0.77748979
71929	1450	control	Unclassified	Gammaproteobacteria	-1.880442664
84598	1348	control	Unclassified	Alphaproteobacteria	-0.598566377
126317	1118	control	Unclassified	Terriglobia	-0.955968099
43546	1805	control	Unclassified	Unclassified	-0.574378676

116435	1162	control	Unclassified	Anaerolineae	-1.131276461
98268	1258	control	Unclassified	Gammaproteobacteria	-0.271086071
99105	1253	control	Unclassified	Anaerolineae	-0.875451876
122808	1133	control	Unclassified	Unclassified	-1.386483878
128194	1110	control	Unclassified	Anaerolineae	-0.903012181
44890	1780	control	Unclassified	Dehalococcoidia	-1.522832587
301972	716	control	Unclassified	Gammaproteobacteria	-1.238456799
29	22637	control	Unclassified	Unclassified	-0.348104506
213762	858	control	Unclassified	Gemmatimonadia	-0.900833096
45361	1772	control	Unclassified	Unclassified	-0.40196028
25173	2255	control	Unclassified	Terriglobia	-0.817573131

113609	1176	control	Unclassified	Unclassified	-0.713729214
45976	1762	control	Unclassified	Unclassified	-0.44297541
142863	1053	control	Unclassified	Unclassified	-0.666576266
118595	1152	control	Unclassified	Unclassified	-0.201611315
305923	711	control	Unclassified	Gammaproteobacteria	-1.087110632
161358	991	control	Unclassified	Unclassified	-1.343847762
69386	1474	control	Unclassified	Unclassified	-0.277595967
68550	1482	control	Unclassified	Unclassified	-0.685692402
98927	1254	control	Unclassified	Unclassified	-0.381615501
112978	1179	control	Unclassified	Unclassified	-0.765624501
56515	1612	control	Unclassified	Gammaproteobacteria	-0.292735189

46802	1749	control	Unclassified	Unclassified	-0.505829088
169010	968	control	Unclassified	Dehalococcoidia	-0.860732922
136140	1078	control	Unclassified	Alphaproteobacteria	-0.718579596
248344	794	control	Unclassified	Unclassified	-0.721749127
56716	1609	control	Unclassified	Gammaproteobacteria	-0.686126455
125550	1121	control	Unclassified	Alphaproteobacteria	-0.985983746
74576	1427	control	Unclassified	Gammaproteobacteria	-0.35444407
67324	1494	control	Unclassified	Other	-0.45857401
136228	1078	control	Unclassified	Terriglobia	-0.751713905
273207	755	control	Unclassified	Gammaproteobacteria	-0.715389273
130957	1099	control	Unclassified	Gammaproteobacteria	-0.443839951

55341	1626	control	Unclassified	Unclassified	-0.769715595
107203	1208	control	Unclassified	Unclassified	-0.389333974
168652	969	control	Unclassified	Betaproteobacteria	-0.753009902
476193	561	control	Unclassified	Alphaproteobacteria	-0.74122658
53554	1649	control	Unclassified	Terriglobia	-1.010321088
389365	625	control	Unclassified	Gammaproteobacteria	-0.527703211
216742	852	control	Unclassified	Gammaproteobacteria	-0.662091912
38173	1907	control	Unclassified	Unclassified	-0.458805741
155663	1009	control	Unclassified	Alphaproteobacteria	-0.546502146
267889	763	control	Unclassified	Gammaproteobacteria	-0.812062811
7354	3687	control	Unclassified	Unclassified	-0.342274226

98769	1255	control	Unclassified	Unclassified	-0.749612309
334620	678	control	Unclassified	Gammaproteobacteria	-0.694580961
89572	1314	control	Unclassified	Unclassified	-0.344979232
49084	1713	control	Unclassified	Anaerolineae	-1.748759041
456262	574	control	Unclassified	Gammaproteobacteria	-0.694796741
365324	647	control	Unclassified	Terriglobia	-0.457509445
34642	1984	control	Unclassified	Unclassified	-0.43743172
34642	1984	control	Unclassified	Unclassified	-0.43743172
404836	612	control	Unclassified	Alphaproteobacteria	-0.457917351
121261	1140	control	Unclassified	Alphaproteobacteria	-0.432530171
116646	1161	control	Unclassified	Unclassified	-0.743475856

53721	1647	control	Unclassified	Unclassified	-0.515236681
1333	7378	control	Unclassified	Other	-0.413546413
127761	1112	control	Unclassified	Unclassified	-0.252376521
205451	876	control	Unclassified	Anaerolineae	-0.764157665
87563	1327	control	Unclassified	Anaerolineae	-0.858591565
164532	981	control	Unclassified	Alphaproteobacteria	-0.60932315
82727	1362	control	Unclassified	Unclassified	-0.181173725
370311	642	control	Unclassified	Alphaproteobacteria	-0.486837153
255724	782	control	Unclassified	Betaproteobacteria	-0.603872381
205443	876	control	Unclassified	Gammaproteobacteria	-0.503920843
67720	1490	control	Unclassified	Gammaproteobacteria	-0.378596956

107757	1205	control	Unclassified	Gammaproteobacteria	-0.673334907
99175	1253	control	Unclassified	Anaerolineae	-0.224122823
262112	772	control	Unclassified	Gammaproteobacteria	-0.309978608
71532	1454	control	Unclassified	Anaerolineae	-0.777620619
205861	875	control	Unclassified	Unclassified	-1.218216439
155690	1009	control	Unclassified	Dehalococcoidia	-1.404515616
265692	575	shell compost	Unclassified	Other	-0.275022515
179357	939	control	Unclassified	Alphaproteobacteria	-0.115501241
127840	1112	control	Unclassified	Gemmatimonadia	-0.393842287
133128	1090	control	Unclassified	Alphaproteobacteria	-0.344072495

45154	1776	control	Unclassified	Unclassified	-0.294597291
49085	1713	control	Unclassified	Anaerolineae	-0.853174771
49085	1713	control	Unclassified	Anaerolineae	-0.853174771
240485	808	control	Unclassified	Unclassified	-0.176322585
10391	3211	control	Unclassified	Unclassified	-0.258107981
101398	1240	control	Unclassified	Alphaproteobacteria	-0.161569794
62381	1544	control	Unclassified	Unclassified	-0.498479023
62381	1544	control	Unclassified	Unclassified	-0.498479023
10390	3211	control	Unclassified	Unclassified	-0.195110051
59098	1580	control	Unclassified	Anaerolineae	-1.040649504

145360	1044	control	Unclassified	Unclassified	-0.282375837
211539	690	shell compost	Unclassified	Gemmatimonadia	-0.272572463
163058	986	control	Unclassified	Betaproteobacteria	-0.131120449
26831	2197	control	Unclassified	Gammaproteobacteria	0.069060144
98367	1226	shell compost	Unclassified	Unclassified	0.265146171
123918	1128	control	Unclassified	Unclassified	0.55431583
28447	2146	control	Unclassified	Unclassified	0.93768436
100778	1206	shell compost	Unclassified	Unclassified	0.458980484
129073	1012	shell compost	Unclassified	Unclassified	0.599007295
149247	907	shell compost	Unclassified	Clostridia	1.465708667
48320	1908	shell compost	Unclassified	Unclassified	0.380648024
117662	1082	shell compost	Unclassified	Unclassified	2.004627876
117662	1082	shell compost	Unclassified	Unclassified	2.004627876
98469	1225	shell compost	Unclassified	Unclassified	0.237087135

113502	1109	shell compost	Unclassified	Unclassified	0.915775886
98461	1225	shell compost	Unclassified	Unclassified	-0.637209874
41269	2084	shell compost	Unclassified	Unclassified	-0.024223025
100241	1210	shell compost	Unclassified	Unclassified	4.430679336
100241	1210	shell compost	Unclassified	Unclassified	4.430679336
13627	3601	shell compost	Vibrio	Gammaproteobacteria	0.471474856
115157	1098	shell compost	Vibrio	Gammaproteobacteria	0.221477937
112034	1119	shell compost	Vibrio	Gammaproteobacteria	-0.031127236
78921	1415	shell compost	Vibrio	Gammaproteobacteria	-0.234807404
61602	1651	shell compost	Vibrio	Gammaproteobacteria	-0.136530725
74300	1469	shell compost	Zobellella	Gammaproteobacteria	2.274657211
60655	1666	shell compost	Zobellella	Gammaproteobacteria	1.683248176

Table S2: List of significantly enriched protein domains 48 hours after chitin addition (p-value < 1e-20). Total Transcripts include the sum of transcripts across the treatment and control samples. Transcript counts in Treated represents the number of transcripts in the shell compost treated samples containing the corresponding PFAM domain.

PFAM	Name	Description	Transcript Count in Treated	Total Transcripts
PF00501.31	AMP-binding	AMP-binding	1223	1831
PF02875.24	Mur_ligase_C	Muramyl ligase, glutamate ligase	238	291
PF01039.25	Carboxyl_trans	Carboxyl transferase	388	512
PF00849.25	PseudoU_synth_2	RNA pseudouridylate synthase	352	465
PF06628.15	Catalase-rel	Catalase related immune responsive	118	134
PF00165.26	HTH_AraC	Bacterial regulatory helix-turn-helix, AraC	328	432
PF00155.24	Aminotran_1_2	Aminotransferase class I and II	970	1346
PF16113.8	ECH_2	Enoyl-CoA hydratase/isomerase	799	1080
PF04069.15	OpuAC	Substrate binding domain of ABC-type glycine betaine transport system	142	155
PF13302.10	Acetyltransf_3	Acetyltransferase (GNAT)	173	214
PF04851.18	ResIII	Type III restriction enzyme, res subunit	207	253
PF03401.17	TctC	Tripartite tricarboxylate transporter family receptor	610	760

PF02028.20	BCCT	Betaine, carnitine, choline family transporter	107	115
PF02824.24	TGS	TGS domain	220	272
PF03180.17	Lipoprotein_9	NlpA lipoprotein	101	110
PF00198.26	2-oxoacid_dh	2-oxoacid dehydrogenase (catalytic)	455	634
PF04095.19	NAPRTase	Nicotinate phosphorribosyltransferase (NAPRTase) family	65	67
PF00199.22	Catalase	catalase	182	202
PF00271.34	Helicase_C	Helicase conserved C-terminal	631	831
PF20579.1	LapA	LapA	110	119
PF02463.22	SMC_N	RecF/RecN/SMC N terminal domain	344	443
PF01266.27	DAO	FAD dependent oxidoreductase	654	933
PF01554.21	MatE	multidrug/oligosaccharidyl-lipid/polysaccharide (MOP) flippase superfamily	138	159
PF00133.25	tRNA-synt_1	tRNA synthetases class I (I, L, M and V)	498	691
PF01225.28	Mur_ligase	muramyl ligase	182	229
PF08028.14	Acyl-CoA_dh_2	Acyl-CoA dehydrogenase, C-terminal	528	710
PF00275.23	EPSP_synthase	EPSP synthase (3-phosphoshikimate 1-carboxyvinyltransferase)	230	302

PF03553.17	Na_H_antiporter	Na+/H+ antiporter family	134	143
PF02786.20	CPSase_L_D2	Carbamoyl-phosphate synthase L chain, ATP binding	559	734
PF02378.21	PTS_EIIC	Phosphotransferase system, EIIC	191	192
PF00324.24	AA_permease	amino acid permease	381	452
PF00083.27	Sugar_tr	sugar and other transport	314	386
PF02222.25	ATP-grasp	ATP grasp	243	310
PF09680.13	YjcZ_2	family of unknown function	68	69
PF09334.14	tRNA-synt_1g	tRNA synthetases class I (M)	426	566
PF01432.23	Peptidase_M3	Peptidase family M3	203	251
PF00015.24	MCPsignal	Methyl-accepting chemotaxis protein (MCP) signaling	899	1230
PF00725.25	3HCDH	3-hydroxyacyl-CoA dehydrogenase, C-terminal	372	487
PF02771.19	Acyl-CoA_dh_N	Acyl-CoA dehydrogenase, N-terminal	840	1166
PF09917.12	DUF2147	Domain of unknown function	65	67
PF00528.25	BPD_transp_1	Binding-protein-dependent transport system inner membrane component	1029	1482
PF00367.23	PTS_EIIB	phosphotransferase system, EIIB	87	88

PF00905.25	Transpeptidase	Penicillin binding protein transpeptidase	273	362
PF03741.19	TerC	heavy metal resistance	166	189
PF00126.30	HTH_1	Bacterial regulatory helix-turn-helix protein, lysR	984	1217
PF12697.10	Abhydrolase_6	Alpha/beta hydrolase family	403	536
PF00395.23	SLH	S-layer homology protein	608	652
PF00171.25	Aldedh	Aldehyde dehydrogenase family	1511	2061
PF00893.22	Multi_Drug_Res	Small Multidrug Resistance protein	84	91
PF20439.1	SpoIVA_C	Sporulation stage IV protein A, C-terminal	79	80
PF02342.21	TerD	stress response in multiple metal recognition complexes	74	78
PF02421.21	FeoB_N	Ferrous iron transport protein B	281	365
PF00860.23	Xan_ur_permease	permease family	269	286
PF08264.16	Anticodon_1	Anticodon-binding domain of tRNA ligase	306	380
PF12833.10	HTH_18	Helix-turn-helix	480	643
PF00441.27	Acyl-CoA_dh_1	Acyl-CoA dehydrogenase, C-terminal	992	1321
PF13336.9	AcetylCoA_hyd_C	Acetyl-CoA hydrolase, C-terminal	162	194
PF00664.26	ABC_membrane	ABC transporter transmembrane region	232	298

PF00106.28	adh_short	short chain dehydrogenase	1302	2091
PF00440.26	TetR_N	tetracycline resistance repressor	496	626
PF20438.1	SpoIVA_middle	Stage IV sporulation protein A, middle	95	96
PF17876.4	CSD2	cold shock	96	107
PF00465.22	Fe-ADH	Iron-containing alcohol dehydrogenase	343	431
PF01810.21	LysE	LysE type translocator	264	310
PF08245.15	Mur_ligase_M	muramyl ligase middle	339	421
PF00561.23	Abhydrolase_1	Alpha/beta hydrolase family	681	982
PF00724.23	Oxidored_FMN	NADH:flavin oxidoreductase	247	303
PF00289.25	Biotin_carb_N	Biotin carboxylase, N-terminal	300	399
PF09547.13	SpoIVA_ATPase	Stage IV sporulation protein A, ATPase	105	106
PF05175.17	MTS	Methyltransferase small domain	204	248
PF01926.26	MMR_HSR1	50S ribosome-binding GTPase	804	1164
PF03922.17	OmpW	outer membrane protein W	115	132
PF02817.20	E3_binding	e3 binding domain	389	527
PF02785.22	Biotin_carb_C	Biotin carboxylase C-terminal	297	374

PF00392.24	GntR	Bacterial regulatory proteins, gntR family	422	550
PF12806.10	Acyl-CoA_dh_C	Acetyl-CoA dehydrogenase, C-terminal like	208	247
PF12146.11	Hydrolase_4	Serine aminopeptidase, S33	541	724
PF00990.24	GGDEF	Diguanylate cyclase, GGDEF domain	835	1261
PF00364.25	Biotin_lipoyl	Biotin-requiring enzyme	748	1062
PF00206.23	Lyase_1	fumarate lyase	374	510
PF00108.26	Thiolase_N	Thiolase, N-terminal	738	1123
PF13685.9	Fe-ADH_2	Iron-containing alcohol dehydrogenase	291	361
PF00892.23	EamA	EamA-like transporter family	340	449
PF02737.21	3HCDH_N	3-hydroxyacyl-CoA dehydrogenase NAD binding	384	514
PF13603.9	tRNA-synt_1_2	Leucyl-tRNA synthetase	201	252
PF00270.32	DEAD	DEAD/DEAH box helicase	509	692
PF13193.9	AMP-binding_C	AMP-binding enzyme, C-terminal domain	727	1108
PF13520.9	AA_permease_2	Amino acid permease	386	464
PF02770.22	Acyl-CoA_dh_M	Acyl-CoA dehydrogenase, middle domain	896	1202
PF03466.23	LysR_substrate	LysR substrate binding domain	1059	1311

PF00587.28	tRNA-synt_2b	tRNA synthetase class II core domain	377	531
PF10612.12	Spore-coat_CotZ	Spore coat protein Z	62	63
PF01546.31	Peptidase_M20	Peptidase family M20/M25/M40	403	550
PF00375.21	SDF	Sodium:dicarboxylate symporter family	163	176
PF13245.9	AAA_19	AAA+ family 19	162	200
PF00672.28	HAMP	HAMP domain	468	585
PF00005.30	ABC_tran	ABC transporter	2821	4342
PF02729.24	OTCace_N	Aspartate/ornithine carbamoyltransferase	249	329
PF00378.23	ECH_1	Enoyl-CoA hydratase/isomerase	853	1175
PF03061.25	4HBT	Thioesterase superfamily	322	429
PF17936.4	Big_6	Bacterial Ig domain	112	117
PF01391.21	Collagen	Collagen triple helix repeat (20 copies)	209	245
PF00704.31	Glyco_hydro_18	glycosyl hydrolase family 18	120	124
PF13304.9	AAA_21	AAA domain, family 21	623	868
PF00269.23	SASP	Small, acid-soluble spore proteins,	88	89
PF00202.24	Aminotran_3	Aminotransferase class-III	685	970

PF07690.19	MFS_1	Major Facilitator Superfamily	1026	1447
PF06434.16	Aconitase_2_N	Aconitate hydratase 2 N-terminus	175	213

Table S3: List of significantly depleted protein domains 48 hours after chitin addition (p-value < 1e-20). Total Transcripts include the sum of transcripts across the treatment and control samples. Transcript counts in Control represents the number of transcripts in the control samples containing the corresponding PFAM domain.

**PFAM**	**Name**	**Description**	**Transcript Count in Control**	**Total Transcripts**
PF03646.18	FlaG	flagellar protein	97	233
PF00016.23	RuBisCO_large	Rubisco large	142	252
PF00978.24	RdRP_2	RNA dependent RNA polymerase	50	74
PF00404.21	Dockerin_1	Dockerin in cellulosome	129	234
PF02085.19	Cytochrom_CIII	Class III cytochrome C family	163	542
PF09699.13	Paired_CXXCH_1	Doubled CXXCH motif	396	1075
PF04969.19	CS	heat shock recruitment	108	222
PF09242.14	FCSD-flav_bind	Flavocytochrome C sulfide dehydrogenase, flavin binding	46	82
PF17886.4	ArsA_HSP20	heat shock like protein found in ArsA	679	1807
PF02123.19	RdRP_4	Viral RNA-directed RNA polymerase	49	87
PF18962.3	Por_Secre_tail	Secretion system C-terminal sorting domain	523	885
PF00428.22	Ribosomal_60s	60s Acidic ribosomal protein	25	31

PF13372.9	Alginate_exp	alginate export	76	128
PF02472.19	ExbD	Biopolymer transport protein	186	679
PF08770.14	SoxZ	Sulfur oxidation protein	118	232
PF04964.17	Flp_Fap	fimbriae associated protein in pilin	327	437
PF13715.9	CarbopepD_reg_2	carboxypepD_reg-like domain	551	1379
PF00593.27	TonB_dep_Rec	TonB dependent outer membrane receptor	451	1580
PF18046.4	FKBP26_C	protein folding	48	83
PF14522.9	Cytochrome_C7	Cytochrome C7 and related cytochrome C	491	1458
PF08206.14	OB_RNB	Ribonuclease B OB domain	321	918
PF02201.21	SWIB	putative p53 associated protein	55	107
PF16371.8	MetallophosN	Calcineurin-like phosphoesterase, N-terminal	40	53
PF13937.9	DUF4212	Domain of unknown function	83	189
PF07596.14	DUF1559	Domain of unknown function	144	207
PF02433.18	FixO	Cytochrome C oxidase, mono-heme subunit	146	348
PF00114.22	Pilin	pilin bacterial filament	236	414
PF00313.25	CSD	Cold shock DNA-binding protein	530	1271

PF09430.13	EMC7_beta-sandw	ER membrane protein complex subunit 7, beta-sandwich	78	172
PF12139.11	APS-reductase_C	Adenosine-5'-phosphosulfate reductase beta subunit	170	382
PF00120.27	Gln-synt_C	glutamate synthetase, catalytic domain	256	913
PF00808.26	CBFD_NFYB_HMF	Histone-like transcription factor (CBF/NF-Y) and archaeal histone	32	36
PF07980.14	SusD_RagB	starch utilization system for glycan uptake	97	263
PF05538.14	Campylo_MOMP	Campylobacter major outermembrane protein	65	88
PF07963.15	N_methyl	2-oxoadipate dioxygenase/decarboxylase	855	1736
PF13609.9	Porin_4	Gram negative porin	958	1576
PF00011.24	HSP20	heat shock 20 family	748	2039
PF01077.25	NIR_SIR	Nitrite and sulphite reductase 4Fe-4S	254	649
PF00101.23	RuBisCO_small	Rubisco small	69	146
PF13620.9	CarboxypepD_reg	Carboxypeptidase regulatory-like	630	1907
PF09361.13	Phasin_2	phasin protein, granule associated	157	419
PF18201.4	PIH1_CS	PIH1 CS-like domain	43	53
PF07715.18	Plug	TonB-dependent receptor plug domain	540	1721

PF13570.9	PQQ_3	Beta-alanine-activating enzyme, beta-propeller	183	593
PF11839.11	Alanine_zipper	Alanine-zipper, major outer membrane lipoprotein	63	136
PF13442.9	Cytochrome_CBB3	Cytochrome C oxidase, cbb3-type, subunit III	581	2323
PF14686.9	fn3_3	Polysaccharide lyase family 4, domain II	128	271
PF00963.21	Cohesin	Cohesin interaction for cellulosome formation	117	196
PF00022.22	Actin	Actin	129	377
PF04077.15	DsrH	DsrH like protein	68	165
PF09694.13	Gcw_chp	Conserved bacterial protein of unknown function	177	263
PF18205.4	VPDSG-CTERM	VPDSG-CTERM motif	87	100
PF20071.2	DUF6467	Domain of unknown function	1141	2786
PF00729.21	Viral_coat	viral coat protein S domain	75	113
PF00496.25	SBP_bac_5	Bacterial extracellular solute-binding protein	541	2125
PF00543.25	P-II	Nitrogen regulatory protein P-II	344	749
PF02788.19	RuBisCO_large_N	Rubisco large N terminal	108	179
PF04744.15	Monooxygenase_B	Monooxygenase subunit B protein	73	77
PF00680.23	RdRP_1	Viral RNA-dependent RNA polymerase	140	259

PF14881.9	Tubulin_3	Tubulin	25	27
PF13860.9	FlgD_ig	FlgD Ig-like domain	379	833
PF01011.24	PQQ	Glucose/ethanol/alcohol dehydrogenase, beta-propeller domain	223	751
PF13458.9	Peripla_BP_6	periplasmic binding protein	500	2211
PF00125.27	Histone	Core histone H2A/H2B/H3/H4 domain	47	52
PF03460.20	NIR_SIR_ferr	Nitrite/Sulfite reductase ferredoxin-like	243	572
PF00118.27	Cpn60_TCP1	TCP-1/cpn60 chaperonin family	1045	3024
PF16732.8	ComP_DUS	Type IV minor pilin ComP, DNA uptake sequence receptor	78	197
PF14339.9	DUF4394	Domain of unknown function	37	49
PF17210.6	SdrD_B	SdrD B-like domain	226	608
PF04358.16	DsrC	DsrC like protein - dissimilatory sulfite reductase	130	349
PF07382.14	HC2	Histone H1-like nucleoprotein HC2	146	202
PF03951.22	Gln-synt_N	Glutamine synthetase, beta-Grasp domain	204	613
PF17802.4	SpaA	Prealbumin-like fold domain	127	340
PF14905.9	OMP_b-brl_3	Outer membrane protein beta-barrel family	119	372

PF13501.9	SoxY	Sulfur oxidation protein	119	237
PF00216.24	Bac_DNA_binding	Bacterial DNA-binding protein	312	1145
PF16842.8	RRM_occluded	Occluded RNA-recognition motif	54	85
PF14537.9	Cytochrom_c3_2	cytochrome c3	360	1103
PF01478.21	Peptidase_A24	Type IV leader peptidase family	86	217
PF06841.15	Phage_T4_gp19	T4-like virus tail tube protein gp19	57	112
PF00669.23	Flagellin_N	Bacterial flagellin N-terminal helical region	483	1670
PF17228.5	SGP	Sulfur globule protein	80	127
PF00034.24	Cytochrom_C	cytochrome C	531	2091
PF00076.25	RRM_1	RNA recognition motif	187	527
PF04896.15	AmoC	Ammonia monooxygenase methane monooxygenase, subunit C	65	68
PF03953.20	Tubulin_C	Tubulin	62	65
PF03863.16	Phage_mat-A	Phage maturation protein	73	176
PF19572.2	PorV	Type IX secretion system protein PorV	63	130
PF03063.23	Prismane	Prismane/CO dehydrogenase family	216	481
PF00267.24	Porin_1	Gram negative porin	444	755

PF00073.23	Rhv	picornavirus capsid protein	58	120
PF00909.24	Ammonium_transp	Ammonium transporter family	368	839
PF02461.19	AMO	Ammonia monooxygenase/methane monooxygenase, subunit A	81	83
PF03143.20	GTP_EFTU_D3	Elongation factor Tu C-terminal domain	396	1657
PF02530.17	Porin_2	porin subfamily	307	356
PF13433.9	Peripla_BP_5	periplasmic binding protein	398	1717
PF06348.14	DUF1059	Domain of unknown function	37	60
PF15511.9	CENP-T_C	Centromere kinetochore component CENP-T histone fold	21	23
PF02635.18	DrsE	intracellular small protein involved in sulfur reduction	247	665
PF13585.9	CHU_C	CHU_C Type IX secretion signal domain	271	460
PF01618.19	MotA_ExbB	MotA/TolQ/ExbB proton channel family	228	858
PF19516.2	DUF6049	Domain of unknown function	19	20
PF00166.24	Cpn10	Chaperonin 10 Kd subunit	462	1387
PF13360.9	PQQ_2	Outer membrane protein assembly factor BamB	223	711
PF00998.26	RdRP_3	Viral RNA dependent RNA polymerase	103	152

PF13179.9	DUF4006	Domain of unknown function	31	41
PF10518.12	TAT_signal	TAT (twin-arginine translocation) pathway signal sequence	379	1065
PF00700.24	Flagellin_C	Bacterial flagellin C-terminal helical region	455	1554
PF05124.15	S_layer_C	S-layer like family, outer domain	33	38
PF16982.8	Flp1_like	S-layer like family, outer domain	65	73
PF07589.14	PEP-CTERM	PEP-CTERM motif	1216	1510
PF07642.14	BBP2	Putative beta-barrel porin-2, OmpL-like. bbp2	105	183
PF13370.9	Fer4_13	4Fe-4S single cluster domain of Ferredoxin	121	335

Table S4: Read metrics for each sample. The total number of reads generated for both metagenomic and metatranscriptomics samples are shown. The number of reads mapped refers to the number of read pairs for each sample that mapped to the metatranscriptomic assembly generated with RNA reads from 48 hours. The read mapping ratios are also displayed. Each technical replicate is also listed, denoted as replicate A or B.

Treatment	Time (Hours)	Replicate	Number of DNA reads mapped	Total Number of DNA reads	DNA reads mapped ratio	Number of RNA reads mapped	Total Number of RNA reads	RNA reads mapped ratio
Shell Compost	0	A	9529805	37748822	0.252453043	112625566	119035600	0.946150278
Shell Compost	0	B	35227289	73548066	0.478969617	130753459	138416684	0.944636551
Control	0	A	12475273	49869718	0.25015728	308883672	325795804	0.94808978
Control	0	B	50677765	106131928	0.477497827	108002956	113099040	0.954941404
Shell Compost	1	A	90407539	216495480	0.417595504	145404509	152082602	0.95608904
Shell Compost	1	B	15092485	35162988	0.429215088	130585269	136523364	0.956504917
Control	1	A	53187276	131523306	0.404394306	116881915	121355058	0.963140037
Control	1	B	21500786	47291988	0.454639082	139731331	146194500	0.955790615
Shell Compost	4	A	48705520	109233612	0.445884001	120194139	126060424	0.953464499
Shell Compost	4	B	37913717	78793740	0.481176766	112616225	118430268	0.950907457
Control	4	A	36367737	88755730	0.409750863	132960863	141018918	0.94285834
Control	4	B	29571969	70098212	0.421864812	121839001	130111840	0.936417477
Shell Compost	8	A	11864825	29792444	0.398249469	121881815	127568622	0.955421585
Shell Compost	8	B	9394796	35839436	0.26213571	123229179	128626708	0.958037261
Control	8	A	10495232	25771998	0.407233929	121426661	126041374	0.963387316
Control	8	B	8836885	35824156	0.24667392	134165447	140197232	0.956976433
Shell Compost	24	A	16179103	37840432	0.427561266	130001005	135391514	0.960185769
Shell Compost	24	B	16498627	32600194	0.506089841	142890233	148861546	0.9598868
Control	24	A	15233730	37817422	0.402823069	144378238	152986444	0.94373223
Control	24	B	17096742	41386148	0.413103002	133664044	140330156	0.952496939

Shell Compost	48	A	43640301	70215438	0.621520028	567748790	574672586	0.987951755
Shell Compost	48	B	26229982	44007816	0.596030078	478614815	485110824	0.986609227
Control	48	A	44223055	92747240	0.476812625	433986271	455399140	0.952979997
Control	48	B	56742518	117870654	0.481396481	466832715	488574780	0.955499003
